# Protein Clustering and RNA Phylogenetic Reconstruction of the Influenza a Virus NS1 Protein Allow an Update in Classification and Identification of Motif Conservation

**DOI:** 10.1371/journal.pone.0063098

**Published:** 2013-05-07

**Authors:** Edgar E. Sevilla-Reyes, David A. Chavaro-Pérez, Elvira Piten-Isidro, Luis H. Gutiérrez-González, Teresa Santos-Mendoza

**Affiliations:** 1 Departamento de Investigación en Enfermedades Infecciosas, Instituto Nacional de Enfermedades Respiratorias Ismael Cosío Villegas, Mexico City, Mexico; 2 Departmento de Virología y Micología, Instituto Nacional de Enfermedades Respiratorias Ismael Cosío Villegas, Mexico City, Mexico; 3 Departmento de Inmunología, Instituto Nacional de Enfermedades Respiratorias Ismael Cosío Villegas, Mexico City, Mexico; Plymouth University, United Kingdom

## Abstract

The non-structural protein 1 (NS1) of influenza A virus (IAV), coded by its third most diverse gene, interacts with multiple molecules within infected cells. NS1 is involved in host immune response regulation and is a potential contributor to the virus host range. Early phylogenetic analyses using 50 sequences led to the classification of NS1 gene variants into groups (alleles) A and B. We reanalyzed NS1 diversity using 14,716 complete NS IAV sequences, downloaded from public databases, without host bias. Removal of sequence redundancy and further structured clustering at 96.8% amino acid similarity produced 415 clusters that enhanced our capability to detect distinct subgroups and lineages, which were assigned a numerical nomenclature. Maximum likelihood phylogenetic reconstruction using RNA sequences indicated the previously identified deep branching separating group A from group B, with five distinct subgroups within A as well as two and five lineages within the A4 and A5 subgroups, respectively. Our classification model proposes that sequence patterns in thirteen amino acid positions are sufficient to fit >99.9% of all currently available NS1 sequences into the A subgroups/lineages or the B group. This classification reduces host and virus bias through the prioritization of NS1 RNA phylogenetics over host or virus phenetics. We found significant sequence conservation within the subgroups and lineages with characteristic patterns of functional motifs, such as the differential binding of CPSF30 and crk/crkL or the availability of a C-terminal PDZ-binding motif. To understand selection pressures and evolution acting on NS1, it is necessary to organize the available data. This updated classification may help to clarify and organize the study of NS1 interactions and pathogenic differences and allow the drawing of further functional inferences on sequences in each group, subgroup and lineage rather than on a strain-by-strain basis.

## Introduction

Influenza A virus (IAV) can infect multiple animal species, including birds and mammals, but it is recognized that the avian species are possibly the natural host. IAV is an enveloped (−) single-strand RNA virus with a genome composed of eight segments that can be exchanged to form novel genomic “constellations” or reassortants [1]. The large diversity observed in IAV [2], [3] is caused not only by reassortment but also by genetic drift, selective pressures, host adaptation, phylogeography, and transient gene hitchhiking [4], as well as other factors.

The most diverse IAV genes, HA and NA, have been divided into 17 and 10 subtypes, respectively [5], [6]. Differences in sequence motifs have been distinguished between subtypes, for example, in glycosylation [7] or hemadsorption activity [8]. Furthermore, host-specific lineages (Equine 1 and 2, Gull, classic and Eurasian swine, Human and various avian lineages) were early identified in HA, NA and other segments, although there is limited phylogenetic congruence between each segment [1].

The third major contributor to IAV diversity is the NS gene, which encodes the NS1 and NS2/NEP proteins [2], [20]. The major role ascribed to the NS1 protein is the antagonism of the host immune response, specifically by inhibition of retinoic acid-inducible gene I (RIG-I) and blockage of the induction of type I interferons [9]–[11]. Nevertheless, NS1 has many other functions to facilitate efficient virus replication and propagation, many of which are normally described on a strain-specific basis [12]–[15]. NS1 is functionally divided into domains: the amino-terminal RNA-binding domain contains amino acids 1–73, while amino acids 74 onwards constitute the effector domain (ED) that mediates interactions with host proteins [16]. The carboxy-terminal domain can vary significantly in length, leaving the protein with 217, 219, 230 or 237 amino acid residues in most known viruses [17].

Early phylogenetic analyses of the NS genes of influenza A led to its classification into two groups (alleles) only: A, for avian and mammalian viruses, and B, for avian viruses mostly [18], [19]. NS1 sequences appear also to group in host-driven lineages [1], including those discovered together with HA subtype 16 found in black headed gulls [5]. More recent efforts to analyze the genetic mechanisms of diversity and the phylogenetics of NS sequences have focused on host-based sequence selection [3], [20]–[22].

Considering NS1 diversity and that genetic segments can cross the interspecies barrier, as has occurred with swine-origin NS1 from A(H1N1)pdm09 virus, it would be practical to apply a numerical nomenclature system, instead of using hosts or locations. This system is similar to the one that is in use for the HA and NA subtypes, which has been applied more recently to follow the evolution of HA in HPAI H5N1 [23].

In this work, we summarize NS1 diversity without arbitrary sequence removal or host or geographical location bias, by clustering the currently available protein sequence data and combining it with maximum likelihood phylogenetic RNA reconstruction and consensus WebLogo comparisons. We propose an updated classification of the IAV NS1 protein into groups, subgroups and lineages, each with characteristic phenetic features.

## Materials and Methods

### NS1 Databases

All full-length IAV NS complete sequences (including NS1 and NS2/NEP) collected from 1902 to July 2012 were retrieved from the NCBI Influenza Virus Sequence Database [24]. For database consistency, the following sequences were removed: incompletely sequenced NS segments, missing either the coded NS1 N-terminus or the coded NS2 C-terminus; those sequences with early NS1 stop codons leaving products with less than 200 amino acids; and those sequences with internal deletions of more than 30 nucleotides or low quality (multiple N’s). After removal, the database consisted of 14,716 nucleotide sequences (*db14716*). Sequence alignments were conducted in MAFFT alignment software version 6.388b [25] and were manually improved in MEGA5 software [26]. In order to analyze NS1 diversity without requiring large computational power and to reach a dataset of manageable size, the database sequences were translated into their corresponding proteins (pdb14716), and the CD-HIT web server [27] was used. Briefly, CD-HIT applies a greedy incremental clustering process on the results of short word similarity filtering, forming clusters of sequences at and above the given similarity threshold [28]. The removal of identical NS1 sequences (similarity = 1) left 4,538 unique parental protein sequences (database pdb4538, which was later reduced to pdb4535 due to three putative recombinants, as explained in Results and pdb4535 was used for analyses). Further sequence clustering to the most similar cluster at or above 96.8% amino acid similarity was conducted considering a global sequence alignment and other default parameters, which produced 415 clusters (pdb415), such that all members in each cluster had at most eight amino acid differences with the cluster representative. In every reduced database, care was taken that the earliest (oldest) sequence in each cluster was designated as the cluster representative sequence, and when changes in size were observed, the earliest size variants were also added as separate clusters. No sequence was removed randomly. This algorithm favors conservation of parental sequences without making assumptions concerning hosts, geographical location of sampling or associations to glycoprotein subtypes. These databases are available from the authors by request. Consensus database sequence logos were built in WebLogo software version 2.8.2 [29].

### Phylogenetic Analyses and Distances

Substitution models for nucleotide sequences were evaluated in MEGA5 and those with the lowest BIC (Bayesian Information Criterion) were selected. The nucleotide sequences corresponding to those proteins in pdb415 were retrieved (*db415*) and used for maximum likelihood phylogenetic reconstruction under a GTR+I+Γ4 substitution model with a bootstrapping value of 500 in MEGA5 [26]. For distance calculations, the Gamma distribution parameter values α = 0.9304 and 1.0587 were used for average pairwise estimations by maximum composite likelihood for nucleotide sequences and by the JTT model for amino acid distances, respectively, in MEGA5. To manage gaps under these analyses, the partial deletion option with a threshold of 50% was considered.

### Detection of Recombinant Events

The recombination detection methods GENECONV, BootScan, MaxChi, Chimaera and 3Seq implemented in the Recombination Detection Program (RDP) version 3.44 [30] were used to analyze discordant sequences.

## Results

### NS1 Phylogenetic Reconstruction

**Figure 1:**
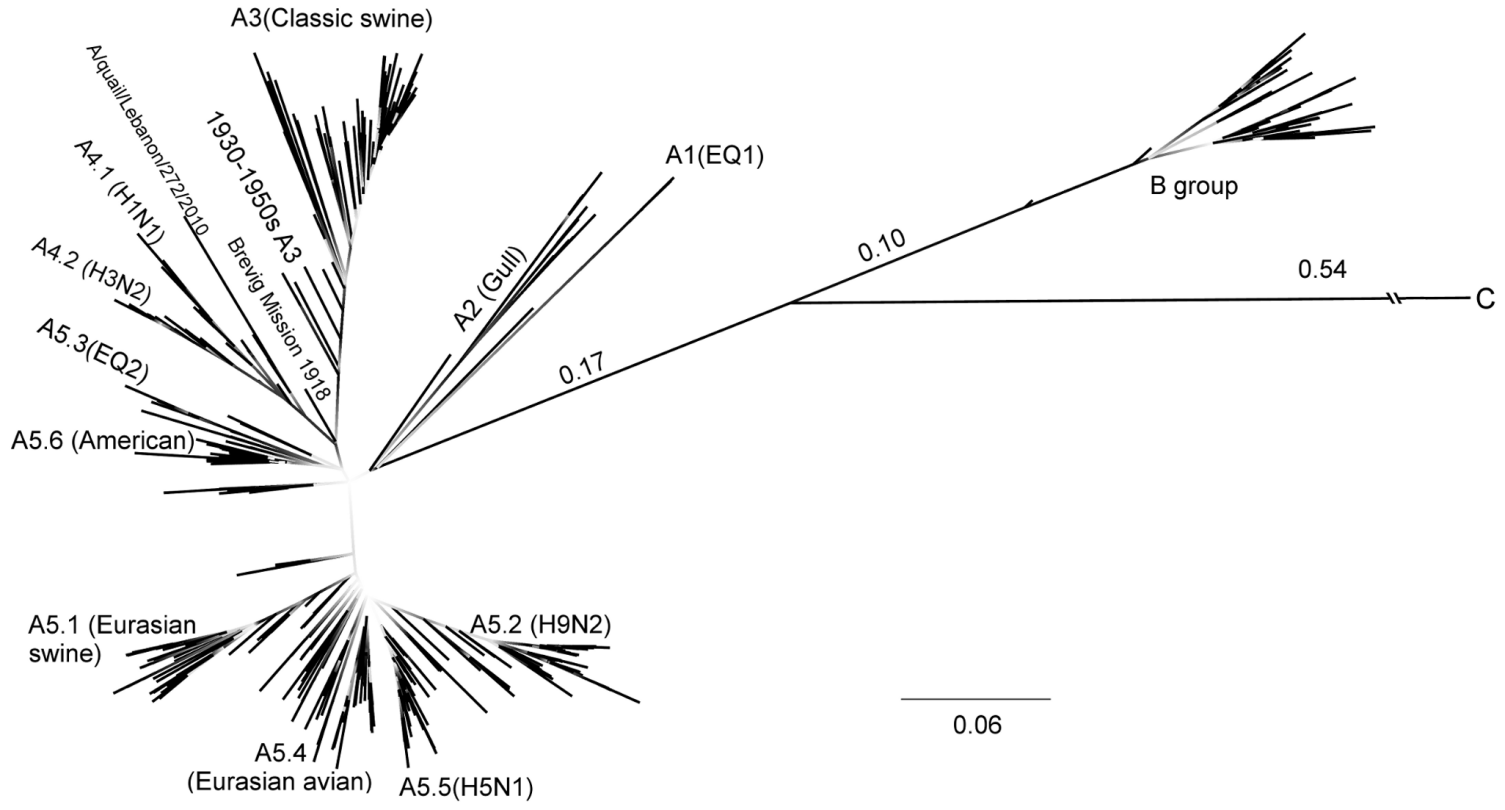
Maximum likelihood phylogenetic reconstruction of NS1-coding RNA sequences using *db415* (415 cluster-representative sequences). A GTR+I+Γ4 model was used to estimate evolutionary rate differences among sites with a bootstrap value of 500. The tree with the highest likelihood was visualized using FigTree 1.3.1 (http://tree.bio.ed.ac.uk/software/figtree/). Darker and thicker lines correlate with higher bootstrap values in branches (>50%). Nodes with the lowest bootstrap values (0.1 or less) were intentionally left white to emphasize uncertainty. The H1N1 A/Brevig Mission/1918 sequence fitted closer to the (A3, A4) node. A1_(EQ1)_ and A2_(Gull)_ share many sequence features with A5_(Avian)_, but their long branches and distances (Table 2) granted them recognition as different A subtypes.

A simple clustering approach was applied to reduce sequence data to a manageable level while ensuring that it still represented the diversity of NS1. The NS1 RNA sequences of those viruses in pdb415 were recovered (*db415)* and used for the maximum likelihood phylogenetic reconstruction of influenza A NS1 in MEGA5 [26]. Overall, the nucleotide tree (Figure 1) had high bootstrap outcome values, supporting the existence of the well-known split between NS1 groups A and B, with the novel NS1 sequences from the provisionally named H17 bat viruses [6] as a more distant group, which we provisionally named NS1 group C. Within group A, multiple branches can be distinguished with an apparent star-like structure, although there was low statistical support (bootstrap) to describe how most of these branches were related. To avoid confusion with hosts, locations and glycoprotein subtypes, we numbered A subgroups according to nucleotide and protein distances (Table 1). As shown before [1], Equine 1 NS1 sequences (EQ1), including A/equine/Prague/1/1956, form the most distant cluster (subgroup) within group A, so we named these A1 subgroup, even though no novel members of this cluster have been identified after 1966. The A2 subgroup is composed of distinct gull sequences forming a complex of long-branch taxa, including A/sabines_gull/Alaska/296/1975 and the NS1 sequences in H16 viruses [5]. There was strong support for branches separating classic swine virus NS1 sequences from those in humans. We named these subgroups A3_(Classic swine)_ and A4_(Human)_, and as shown, we provide here the commonly used name as a subscript in parenthesis only for clarification purposes. Two distinctive branches within A4 prompted the distinction of lineages A4.1_(seasonal H1N1)_ and A4.2_(seasonal H3N2)_. NS1 in human H2N2 virus sequences appear as common ancestors of NS1 from H3N2 (A4.2), rather than as predecessors of H1N1 (A4.1). The rest of the sequences were included in subgroup A5 (Figure 1), forming further lineages that we, based on nucleotide sequences, named A5.1_(Eurasian swine)_, A5.2 _(H9N2)_, A5.3_(EQ2)_, A5.4_(Eurasian avian)_, A5.5_(H5N1)_ and A5.6_(American avian)_. Interestingly, the A5.4 and A5.6 branches are indistinguishable by amino acid sequence (Figure S1), but they can be distinguished by nucleotide sequence, with A5.4 sequences having a T or C in nucleotide position +126, G or A in +180 and T in +222. By contrast, A5.6 sequences display no nucleotide restriction in +126, T or C in +180 and mainly C with a low frequency of T in +222. Of note, NS1 A/Brevig_Mission/1918 was located close to the A3, A4 branching node in the RNA phylogeny (Figure 1) but encodes a protein closer to the A5 sequences by retaining the sumoylation residues, the CPSF30 and the crk/crkL-binding motifs (see below), while having KSEV as a characteristic PDZbm (PDZ domain binding motif).

**Table 1:** Distance estimation within classes for the novel NS1 classification using nucleotide and protein sequences.

			Average distance within class	
Group	Subgroup	Lineage	Nucleotide	Aminoacid
A			0.173	0.180
	A1		0.024	0.030
	A2		0.112	0.099
	A3		0.055	0.070
	A4		0.073	0.092
		A4.1	0.037	0.055
		A4.2	0.026	0.039
	A5		0.102	0.100
		A5.1	0.058	0.079
		A5.2	0.064	0.071
		A5.3	0.026	0.047
		A5.4	0.066	0.070
		A5.5	0.036	0.051
		A5.6	0.042	0.029
B			0.076	0.050
C			0.036	0.013

Model selection with the lowest BIC value and distance estimations were conducted in MEGA5 [26]. Gamma distribution parameter value α = 0.9304 and 1.0587 were used for maximum likelihood distance estimations using the GTR+I+Γ4 and the JTT+Γ4 models for nucleotide (*db4535*) and amino acid (pdb4535) substitutions, respectively. Within class distances for unique NS1 nucleotide and protein sequences, A1 corresponds to Equine 1 sequences, A2 to gull sequences, A3 includes viral sequences grouped under classic swine, A4 includes all former human seasonal viruses with A4.1 comprising H1N1 sequences and A4.2 including H3N2 and H2N2. Within A5 are A5.1 (Eurasian swine virus sequences), A5.2 (sequences associated with H9N2 and H6N1 viral sequences from Pacific Ocean avian species), A5.3 (Equine 2 sequences), A5.4 (Eurasian avian), A5.5 (HPAI H5N1) and A5.6 (American avian). Identification of highly distant sequences within each class (by observation of intraclass pairwise comparison matrices) resulted in sequence relocation and some iteration was conducted to reach robust A5 lineages.

### NS1 Classification

The average pairwise distances for nucleotide and amino acid sequences were calculated within divisions (Table 1) and between divisions (Table 2). The distances within group A were more than double of that in group B, while there is limited significance concerning the distance values in group C as they were calculated from two sequences only. Distances within A subgroups were equal or below 0.112, and the A2 sequences as the most diverse, as they formed the long-branch cluster observed in the phylogeny (Figure 1). A4 and A5 lineages had discrete intrasubgroup distances values below 0.080. When comparing between groups, B and C were highly distant from any of the A subgroups. The cut-off distance value between the subgroups appears to be 0.2, both with nucleotide and amino acid sequences.

**Table 2:** Matrix of nucleotide and protein distances between NS1 groups, subgroups and lineages in *db4535* and pdb4535, respectively.

	A1	A2	A3	A4.1	A4.2	A5.1	A5.2	A5.3	A5.4	A5.5	A5.6	B	C
**A1**		0.376	0.409	0.364	0.425	0.350	0.320	0.329	0.309	0.329	0.306	0.617	1.500
**A2**	0.331		0.319	0.230	0.281	0.245	0.246	0.226	0.189	0.216	0.179	0.513	1.330
**A3**	0.364	0.314		0.265	0.308	0.275	0.275	0.235	0.219	0.236	0.197	0.519	1.425
**A4.1**	0.293	0.218	0.233		**0.173**	0.222	0.227	0.184	0.146	0.176	0.135	0.482	1.439
**A4.2**	0.329	0.256	0.256	**0.127**		0.282	0.270	0.246	0.201	0.225	0.191	0.454	1.431
**A5.1**	0.299	0.249	0.270	0.200	0.233		**0.201**	**0.183**	**0.142**	**0.176**	**0.129**	0.536	1.306
**A5.2**	0.277	0.252	0.280	0.212	0.225	**0.168**		**0.164**	**0.136**	**0.168**	**0.131**	0.482	1.286
**A5.3**	0.270	0.234	0.228	0.171	0.204	**0.182**	**0.162**		**0.118**	**0.149**	**0.106**	0.514	1.354
**A5.4**	0.250	0.204	0.230	0.149	0.174	**0.127**	**0.118**	**0.126**		**0.095**	**0.053**	0.471	1.328
**A5.5**	0.262	0.224	0.227	0.164	0.179	**0.144**	**0.132**	**0.134**	**0.074**		**0.074**	0.469	1.312
**A5.6**	0.261	0.196	0.201	0.130	0.167	**0.150**	**0.144**	**0.107**	**0.090**	**0.104**		0.463	1.317
**B**	0.720	0.678	0.633	0.645	0.595	0.679	0.662	0.645	0.619	0.627	0.604		1.304
**C**	1.533	1.524	1.636	1.642	1.615	1.415	1.418	1.492	1.426	1.419	1.448	1.321	

The lower left corner corresponds to nucleotide sequences and the upper right corner corresponds to amino acid sequences. Lineages (A4 and A5) are highlighted in bold. Suspected recombinant sequences were excluded from these analyses.

The composition of the unique sequence database (pdb4535) was as follows: A5 (45.32%), A4_(Human)_ (22.07%), A3_(Classic swine)_ (19.54%), B (12.30%), A2_(Gull)_ (0.66%), A1_(Equine1)_ (0.07%) and C (0.04%). Within A5, the most diverse lineage is A5.5 (21.36%), followed by A5.4 (20.97%), A5.6 (16.45%), A5.2 (14.60%), A5.3 (6.18%) and A5.1 (5.55%). In fact A/Brevig_Mission/1918 and other 305 (14.89%) sequences were excluded from A5 specific lineages, due to their location towards bifurcating nodes, but still remaining within A5 boundaries. The unique sequence database was used to produce separate consensus sequences (Figure S1) in the form of sequence logos using WebLogo. Comparisons between sequence logos allowed the identification of thirteen amino acid positions, which can be used for NS1 sequence classification into identified groups (A, B and C) and subgroups (Table 3) with further lineages in A4 and A5 (Table 4). When the thirteen amino acid residues are considered, more than 90% of sequences in most classes are entirely compliant with Table 3. From the rest of sequences most have one mutation only, while two or three mutations are rare (A3: one sequence, A4: three sequences, A5: twelve sequences and B: two sequences) and still remain easily classifiable. Only three sequences in pdb4538 (0.07%) contained incongruent amino acid patterns (outliers) and were analyzed in detail (see below). This classification is unaffected by host species, geographic location or surface glycoprotein association.

**Table 3:** Thirteen amino acid residues used to classify NS1 sequences.

	NS1 amino acid position															
Class	22	25	59	67	81	91	111	116	189	206	215*	217*	227*	Percentage of Class Coverage	Variants (%)	Representative isolates
**A1**	F	Q	R	R	I	T	R	Y	N	S	S	K	G	67		
			L					C						100	0	A/equine/Prague/1/1956
**A2**	F	Q	R	R	I	T	F	C	D	S	A	K	E	57		
			C			I		R			T	E	G	87	4 (13)	A/gull/Maryland/704/1977
**A3**	F	N	L	W	I	S	I	C	G	C	P	E	Gap	47		
	L	S	F	R	V	A	V	Y	D	R	S	stop		86		
		Y					M		S	H				90		
							T			S				91		A/California/7/2009
***(1930–40’s)***		*K*	*R*									*K*	*R*	93	64 (7)	A/Swine/Iowa/15/1930
**A4**	V	Q	H	K	M	T	V	C	D	S	T	K	R	45		
	I	K	C	R	I		M	S	N	N	A	T	Gap	80		
			R	N			A					E		90		A/WSN/1933,
			Y											91	88 (9)	A/Puerto_Rico/8/1934
**A5**	F	Q	R	R	I	T	V	C	D	S	P	K	E	31		
	I	W	H	Q	gap	S	M	S	G	I	S	N	G	63		
	L	R	C	K	V	A	I		N	T	L	D	K	79		
			Y	H	T	I	T			C				90		
			S	W						G				94	128 (6)	A/chicken/Rostock/8/1934
**B**	L	R	M	D	I	T	I	M	D	R	P	K	E	57		
			I	N		C	M	I	N	C	S	N	G	74		
			T	E			V	V		H	L		gap	94	36 (6)	A/chicken/Mexico/232/1994

The most common amino acids (>1% frequency) in *db4535* are listed on each position based on their frequency with the exception of A1, which is composed by three unique sequences only. Because of the large diversity in NS1, it is necessary to apply the whole set to classify sequences without applying phylogenetic methods. The number of sequences covered by the given residues is described as percentages. Those sequences with one or more polymorphisms away from this list are described as variants. A/Fort_Monmouth/1/1947 NS1 is the sequence with the largest number of differences (4) due to a truncation in amino acid 202. Residues in positions 25, 59, 217 and 227 were used to identify early A3 sequences collected between 1930 and 1942 are marked in italics. K26 and V90 are also characteristic of the A1 and A2 subtypes, respectively. The C group was excluded because of its large divergence from the A and B groups and the difficulties with achieving a satisfactory alignment.

* Positions 215, 217 and 227 allow gaps in the A subgroups.

**Table 4:** Characteristic amino acid signatures in the classification of major avian and human host lineages.

Lineage	Characteristic amino acid signatures
A4	
A4.1 H1N1	A23, M98, A112, E229
A4.2 H3N2	V23, L98, E112, K229
A5	
A5.1	G26, K183
A5.2	R55, E60, P87, L103, I106
A5.3	D96, E139, K186, H207
A5.5	Deletion 80–84, K118, P/L212

A5.4 and A5.6 can only be distinguished by nucleotide sequence considering that A5.4 sequences have T or C in nucleotide position +126, G or A in +180 and T in +222, while A5.6 sequences show no nucleotide restriction in +126, T or C in +180 and mainly C with low frequency of T in +222.

### Outliers

**Figure 2:**
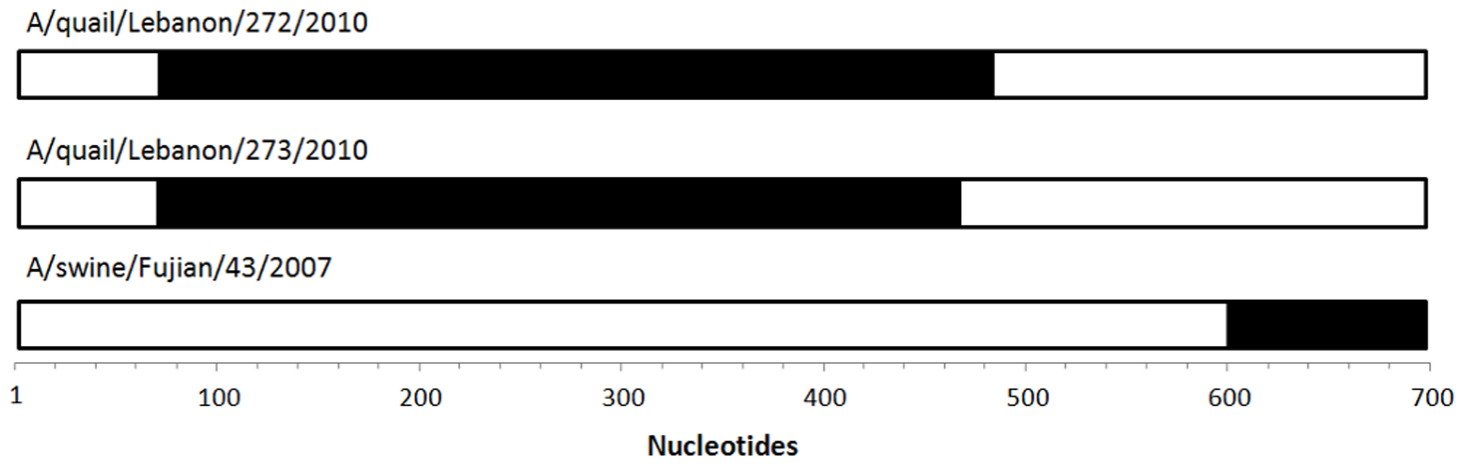
Recombinant detection in three incongruent sequences. The NS1-coding region of Lebanese sequences where two cross-over events were indicative of the introduction of a segment 99% similar to A/Puerto Rico/8/1934 (black boxes) of 425 nucleotides for A/quail/Lebanon/272/2010 and of 389 nucleotides for A/quail/Lebanon/273/2010, within a NS frame segment related to A/duck/Alberta/60/1976 (B group). These events were strongly supported by 3Seq (p = 10^−10^), MaxChi (10^−9^), Chimaera (10^−8^), GENECONV (10^−7^), RDP (10^−6^) and BootScan (10^−4^) methods. In the case of A/swine/Fujian/43/2007, most of the segment is similar to the cluster of A/chicken/Rostock/8/1934 (NS1 A5), but one cross-over event was distinguished by nucleotide 592, introducing a 200-nucleotide region (beyond the end of NS1) 99% similar to A/Puerto Rico/8/1934 and supported by BootScan (p = 10^−6^), GENECONV (10^−4^), MaxChi and Chimaera (10^−3^) methods.

Unclassifiable sequences (A/quail/Lebanon/272/2010, A/quail/Lebanon/273/2010 and A/swine/Fujian/43/2007) were analyzed for within segment recombination together with a representative from various groups and subgroups in RDP software version 3.44 [30]. Well-supported recombination events involved the introduction of nucleotide sequences 99% similar to NS from A/Puerto Rico/8/1934 (A4.1), of 390 nucleotides into the aforementioned Lebanese sequences and of 200 nucleotides into A/swine/Fujian/43/2007 (Figure 2). The high similarities to A/Puerto Rico/8/1934 suggest that laboratory artifacts may have generated these sequences. Further laboratory chimeras, particularly those involving sequences from the same class, may be present in our databases. The presence of recombinant sequences in public databases has been demonstrated previously [31].

### NS1 Size Diversity

**Figure 3:**
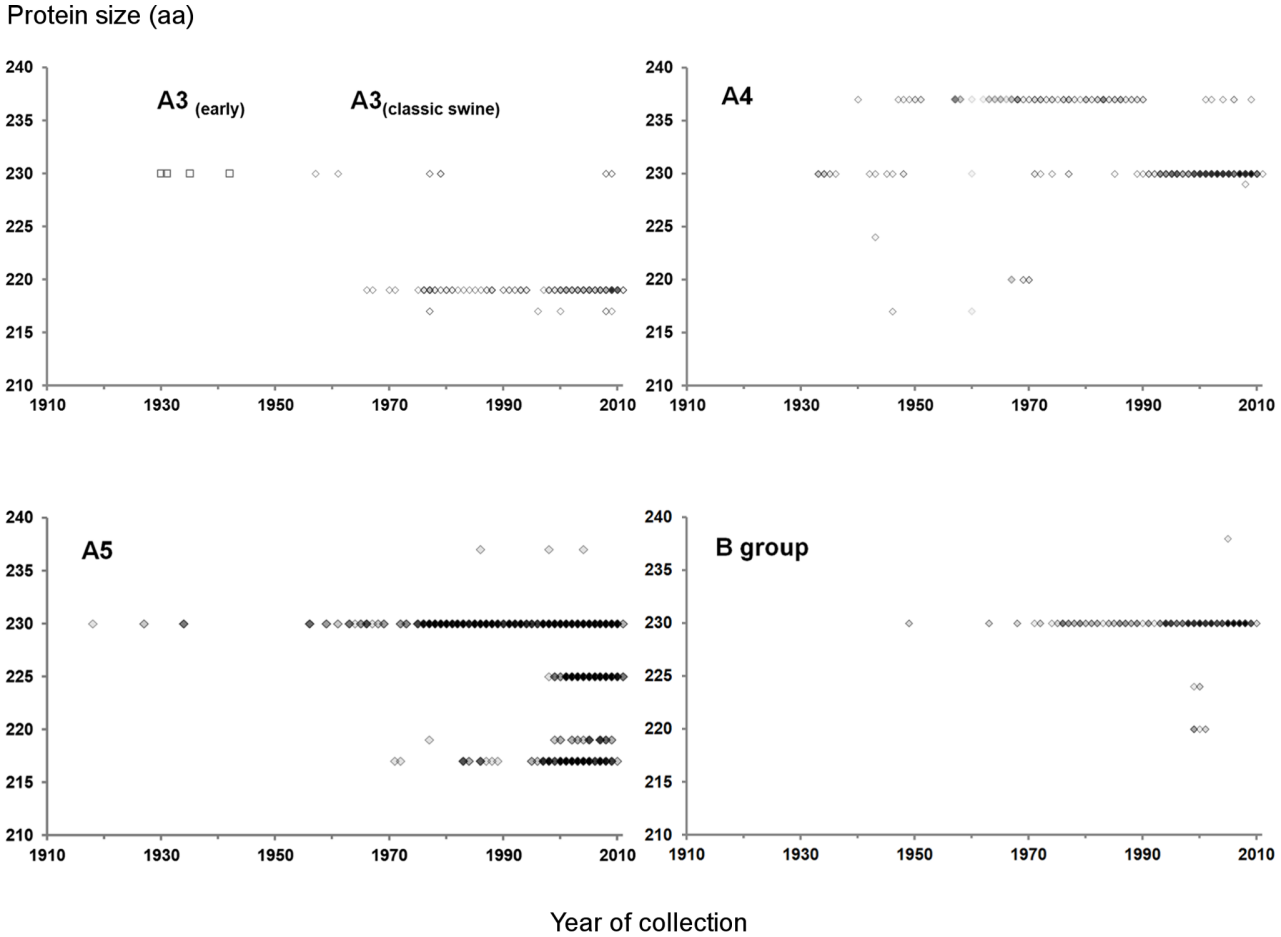
Length diversity of encoded IAV NS1 from 1910 to 2011. Only sequences in *db4535* with canonical NS1 protein sizes were considered. Dot density represents the number of unique sequences with same length at a given time point, i.e., darker dots represent higher sequence diversity regardless of the prevalence of identical sequences. Characteristic patterns of NS1 length can be observed for each A subgroup and the B group. NS1 A5 and B cocirculate in avian species. However, B group sequences maintain a consistent 230aa length, in contrast to A5, which are currently cocirculating in 217, 219, 225 and 230aa versions. A3 sequences have remained at 219 aa, while the most frequent A4 sequences were at 237 aa for most of the second half of the twentieth century, but the 230aa proteins regained prevalence in about 1990. The 225aa length, exclusive to HPAI H5N1 viruses, emerged at the end of the 1990s due to an internal deletion.

Each sequence in *db4535* is the oldest/earliest member of a cluster of identical sequences and was used to study protein length diversity and sequence diversity (Figure 3). NS1 protein diversity can be represented by the number of unique sequences at a given time point, although it is evident that there is higher diversity in more recent years, but this diversity could be associated with more frequent sampling and extended sequencing capabilities. NS1 protein length profiles are unique for each of the divisions presented here, and 230 amino acids (aa) in length is the most frequent protein size in viruses infecting avian species, particularly those in group B. However, A5 sequences can have different protein sizes characteristic of certain lineages. For example, A5.2_(H9N2)_ is mostly (81.33%) 217 aa, opposite to A5.4 in which only 16.24% has 217aa; A5.5_(H5N1)_ has 225 aa due to an internal 5 aa deletion (Figure S1). Most A3 sequences (96.96%) have lost 11 aa at the C-terminus, leaving these proteins with 219 aa. The profile of A4_(Human)_ sequences is also remarkable: 230 aa was apparently the most common length in diversifying A4_(Human)_ sequences during the 1930s; however, a 7aa elongation became fixed both in H1N1 and H3N2 in human viruses in about the 1940s, which eclipsed the C-terminal PDZ-binding motif and remained in diversification until the late 1980s, when viruses with 230 aa regained sequence diversity. Figure 3 also indicates that sporadic outbursts of NS1 variation with “unusual” protein sizes diversify for a short time in all subgroups at a given length. These changes in NS1 protein sizes mostly involve sporadic mutations associated with stop codons and might be unrelated to interspecies transmission or novel segment reassortments. For example, A(H1N1)pdm09 NS1 sequences detected in humans are all in the A3_(Classic swine)_ subgroup with 219 aa, while the sequences from HPAI H5N1 viruses infecting humans are in A5.5.

### Conserved Functional Motifs Patterns

As a consequence of our classification we found several instances of motif sequence conservation within subgroups and lineages (Table 5) present in characteristic patterns (Table S1). As examples, we focused on four functional motifs and analyzed the frequencies of the conserved residues in the non-redundant database (pdb4535), regardless of the representation that each of those sequences might have in larger databases, such as pdb14716.

**Table 5:** High domain conservation in NS1 groups, subgroups and lineages.

		CPSF30bm					crk/crkLbm			Sumoylation				PDZbm			
	Residue	103	106	108	125	189	212	214	215	217	219	221	222	227	228	229	230
		*F*	*M*	*K|R*	*D|E*	*D|G*	*P*	*Φ*	*P*	+ *K*	*K*	*K*	*M*				
A subgroups																	
A1		Y|F	I	N	D	N	F	L	S	K	K	K	M	G	P	E	V
A2		F	M	K	D	D	P	F	A|T	K|E	E	K	M|L|V	E	S	E	V
A3		F	M	R	E	G	P	L	P	E	K	–	–	–	–	–	–
A4	A4.1	F	M	K	D	D	P	F|L	T	T|K	K	K	M	R	S	E	V
	A4.2	F	M	K	E|D	D	P	L	T	K	K	K|E	M	R	S	K	V
A5	A5.1	F	M	K	D|E|N	D|G	P	F|L	P	K	K	E|K	M	G	P	K|E	V
	A5.2	L	I	K	D	D	P|S	L	S|P*	K	(K)	(K)	(M)	(E)	(P)	(E)	(V)
	A5.3	F	M	K	D	D	P	F|L	P	K	K	K	M	K|E	P|S	E	I|V
	A5.4	F	M|I	K	D	D	P	L|F	P	K	K	K	M	E	S|P	E	V
	A5.5	F	M	K	D	D	L|P	L|F	P|L	N|D	K	K	M	E	S	E|K	V
	A5.6	F	M	K	D	D	P	L	P	K	K	K	M	E	S	E	V
B group		Y	M	R	D|N	D	P	L	P|S	K	K	Y	M	E	S	E|K	V|I

Pattern of prevalent (at least 85%) amino acid residues in indicated positions for each class in *db4535*. The motif conservation (%) is described in the Results section. F103 and M106 are indispensable for CPSF30 binding [33], [34]; FMKDD motif results in strong binding, while FMREG results in weak binding [32]. Prolines in 212 and 215 as well as positive (K) aa in 217 residues are indispensable for crk/crkL binding [15];

* only 4.33% of sequences have both P212 and P215 in A5.2, while letters in parentheses represent consensus residues for 230aa sequences (18.67%). Lysines 217, 219 and 221 are susceptible to sumoylation, while M222V mutation inhibits sumoylation [35]. Amino acids 227–230 constitute a PDZbm [2]. The bar (|) notation represents the most common residues in that position in decreasing order of frequency.

### Binding of NS1 to CPSF30

Structural and reverse genetics results focused on the CPSF30:NS1 interaction [32]–[34] have shown that NS1 F103 and M106 residues are indispensable for the NS1:CPSF30 interaction [33], [34], in addition to other residues (K108, D125, D189) to increase affinity [32]. The FMKDD motif is present in most sequences in A2 (96.67%), A4.1 (88.04%), A5.3 (98.43%), A5.4 (83.76%), A5.5 (95.67%) and A5.6 (96.45%). In addition, 83.77% of A4.2 sequences have the FMKED motif, which may display mild interactions with CSPF30. The FMKDD and FMKED motifs are not present in A3, while 86.34% of the sequences in this group have the FMREG motif; hence, these sequences may display weak interactions with CPSF30 [32]. Most NS1 sequences classified as A1 (100%), A5.2 (99.67%) and B (99.46%) have other motif sequences that may block the interaction (Table 5). This classification reveals high sequence conservation in the CPSF30-binding motif within classes.

### crk/crkL Binding

Avian, but not human viruses, may induce PI3K activation through the interaction of NS1 with crk/crkL adaptor proteins [15]. The interaction is mediated by a consensus class II SH3 domain-binding motif defined by the P*X*ΦP*X* **+** sequence (*X*: any, Φ: hydrophobic and **+**: positively charged amino acids) corresponding to residues 212–217 in NS1. As P212, P215 and K217 residues are indispensable for crk/crkL binding [15], our model suggests that 80.47% of sequences in the B group and 92.02% in the A5 subgroups, without including A5.2 and A5.5 (in which only 4.33% and 1.37% would bind crk/crkL respectively), might bind crk/crkL. A1 (100%), A2 (100%), A3 (98.42%) and A4 (99.90%) sequences have mutations that would block crk/crkL binding (Table 5).

### Sumoylation

NS1 protein stability and early viral replication is associated with sumoylation [35], [36], mostly in K221, but a contribution is recognized also for K217 and K219. The combination of K217 and K219 or the combination of K219 and K221 accounts for strain-specific NS1 sumoylation [35]. Most sequences in A1 (100%), A4 (96.30%), A5 (93.51% considering all lineages except 5.2) and B (93.73%) meet the requirements to be sumoylated (Table 5). The pandemic H1N1 strain A/Sichuan/1/2009 has the K217E mutation and a terminal truncation in K219, features that have been shown to cancel sumoylation [35] and that are present in >95% of A3 sequences, suggesting that these sequences would not be sumoylated. Most A5.2 sequences (76.67%) end with K217. Whether this terminal residue can be sumoylated still needs to be tested, but 230aa A5.2 sequences contain an active sumoylation site. Although this motif is next to the crk/crkL bm and may share residue 217, we observed that A1, A4 and A5.5 lack an active crk/crkL bm but have an active sumoylation motif, suggesting that independent motif evolution may occur. This possibility should be explored in future research.

### PDZ-binding Motif

The PDZ-binding motif (PDZbm) of NS1 (residues 227–230) has been related to pathogenicity and host adaptation [2], [37], [38]. The consensus PDZbm sequence ESEV, which is mostly present in avian viruses, apparently provides more virulence than the consensus RSKV of human viruses [2], [37]. In our model, 98% of A3 and 81.33% of A5.2 sequences lack this domain; the other classes displayed PDZbm class-specific sequences (Table 5). Of note, A4.1 sequences (88.81% of 230aa sequences) bear the RSEV motif, which is different from the RSKV motif present in (95.73% of 230 aa sequences) A4.2 (Figure S1). Most sequences in the A5 subgroup and B group (76.93% and 73.99% of 230aa sequences respectively) have virulent PDZbm ESEV/EPEV [37].

The analysis of these functional motifs highlights their class conservation without making any assumptions concerning host and host adaptation, geography, or glycoprotein association. Furthermore, this analysis reveals a characteristic motif profile for each class (Table S1) and possible independent motif evolution.

## Discussion

The concept of classification in biology has been thoroughly discussed [39]–[41]. It is generally accepted that classifications in science can be of two main types. The first is based on conventions and used to group practical observations, such as phenotypes, hosts or geographic associations. The second is based on theories or models. The phylogeny presented here is of the latter type. In this work, we propose a theoretical classification based on phylogenetic data for the IAV NS1 RNA along with distance estimations in order to classify all available IAV NS1-coded proteins regardless of host, location and date of sampling.

Extensive genetic drift can interfere with phylogenetic reconstructions and may lead to assumptions that such methods may provide scant information relevant to clinical and functional areas [42]. Thus, phenetic analyses have taken the lead in understanding genotype-phenotype correlations. NS1 phenotype analyses based on the coded C-terminus sequence length [17], [43] have identified a number of length variation types (LVT) and recognized that some NS1 sequences sharing the same LVTs may still be distantly phylogenetically related. The main issue with phenetic grouping, for example, by coded size or host, is that limited functional inferences can be drawn and used on a wider number of sequences and in other domains. For Noronha et al. [42] the evolution of a clinically important motif in influenza A virus might be missed when the phylogeny of the segment is considered. On the contrary, in our work, we provide evidence that by using phylogenetic RNA data from clustered protein sequences, we can observe characteristic motif profiles which are highly conserved within the proposed classes. Furthermore, such characteristic motif conservation is maintained when, as an example, we looked at two SFs from Noronha et al. and found characteristic VT profiles (Table 6).

**Table 6:** Distinct functional sequence feature variant types (SF-VT) [42] are characteristic of each NS1 class obtained by RNA phylogenetic reconstruction.

	crk/crkLbm	PKRbm
Division	VT	VT
A1	37	9, 31
A2	20, 21	18, 20, 25, 73
A3	3, 15	1, 2, 10
A4.1	1, 5, 16	1, 13, 15
A4.2	1, 10	1, 8
A5.1	2, 8, 30	1, 3, 9, 24
A5.2	2, 6, 14	1, 3, 5, 7, 11, 12, 14, 22
A5.3	2, 8	1
A5.4	2, 8, 18	1, 3, 26
A5.5	4, 7, 9, 11, 13	1, 3, 6
A5.6	2, 6	1, 9
B	2, 6, 12	4, 9

crk/crkL (Influenza_A_NS1_SF33) and PKR (Influenza_A_NS1_SF30) binding motifs [42], [52] were analyzed as examples, and the numbers represent protein sequences (variant types) as described [52]. The most common VTs (>90% of sequences) are enlisted.

Furthermore, phenetic grouping may also complicate analyses, as IAV can jump to other species as a completely foreign virus, such as in the case of the HPAI H5N1, or as reassortants, as was the case with A(H1N1)pdm09 and its further reassortants [44].

As the NS RNA substitution rates observed in subsets of avian viruses are nearly as high as those from HA or NA [20] and as NS is another major contributor to genetic diversity in IAV [2], a detailed classification may contribute to understanding the role of NS in IAV infection, beyond the NS1 groups A and B recognized for many years [18], [19].

We propose here an algorithm to summarize NS1 diversity, without arbitrary sequence removal or bias by host or geographical location. Different methods for reducing oversampling bias, e.g., due to outbreaks, have been applied, such as removing sequences from the same place and same year [20]. We have addressed oversampling by using an inclusive clustering algorithm that gives equal value to all NS1 variants. Similar approaches have been used mostly for building non-redundant protein databases in UniRef [45] or in deep sequencing protocols for phylotype identification [46]. By using this approach, we generated a database to estimate the maximum likelihood phylogeny of NS1 within reasonable computational time and discerned distinct NS1 variants from the abundant genetic drift. Although giving equal value to all sequences may be another form of bias and although oversampling (outbreaks) is still an artifact omnipresent in publicly available databases, we were able to summarize and analyze all currently known NS1 diversity.

We analyzed the RNA phylogeny of NS1 and observed that a number of classes with shared characteristics could be added as subgroups and lineages to the previously established classification of NS1 [19]. As a result, extensive functional motif conservation was observed within NS1 branches (Table 5), with characteristic profiles for each motif (Table S1). In our paradigm, RNA phylogeny is prioritized, and the phenetic traits are considered afterwards. The method that we present here could be extended to the rest of segments, so that a segment-to-segment nomenclature that relies on sequence phylogenetic reconstruction rather than “recent” phenetic traits (geographical or host transmission history) of a given segment may be implemented. Further studies on NS2/NEP can be conducted using our same databases in the near future.

Because of the large substitution rate of IAV, not all characters are always present in all members of a class (subgroup/lineage), but this limitation falls within the caveats of any classification. A virus species is defined as a polythetic class that constitutes a replicating lineage that tends to occupy a particular ecological niche [47], [48]. In a polythetic class, no character or property is necessary or sufficient to define the class. That is, no grouping is a universal class definable by a single property. By analogy, we used nucleotide/protein distances and phylogeny as instruments to define hierarchical classes, each identified by consecutive decimal numbers (Table 1 and 2; Figure 1).

Our structured numerical classification, unaffected by host shift or reassortment, was organized similarly to the recent H5N1 classification [23] based on phylogenetics and average pairwise nucleotide distances. Thirteen residue positions were required to classify any given NS1 sequence. Certain residues in the same position may be common to two or more polythetic classes. Thus, all 13 positions must be considered to assign each sequence to a class.

Four of the 13 amino acid residues identified in our model of classification (residues 22, 81, 215 and 227) have been recognized as characteristics for human-to-human and avian-to-avian transmission [49], while residue 227 is important in distinguishing human from avian-host viruses [50] when applying different methodological approaches. Whether the other nine residues are important in host range restriction remains to be explored, particularly as other hosts are considered.

Along with particular amino acid substitutions, we can distinguish phylogenetically related sequences with characteristic NS1 size patterns, due mostly to truncations on the C-terminal domain (Figure 3). The biological implications of these variations are beyond the aim of this paper, but they may be related to fitness peaks (see below) and interactions with other viral and host biomolecules (epistasis).

Similar to HA or NA, NS1 shifts should be considered after large sequence differences, mainly due to the acquisition of a NS segment from a different group, subgroup or lineage, as well as the respective phenotypic change [14], [19], [51]. Balancing selection appears to limit NS1 pool diversity, which is supported by the absence of “intermediate” protein types [21], a limited number of NS1 size variants [17], and the existence of the characteristic groups, subgroups and lineages observed in this work, each probably representing fitness peaks for NS1 [21]. The existence of these fitness peaks is supported by preliminary results suggesting that the non-synonymous substitution rate within NS1 A subgroups and lineages is similar, but the synonymous substitution rates vary extensively, particularly in A5 and B (publication in preparation).

Previous work indicated a lack of genetic linkage for the NS segment with the rest of segments in IAV [21], but our work suggests that this may not be the case. For example, in pdb4535 all NS1 A4.1 sequences are associated with human H1N1 segments, and most A4.2 are associated with H3N2 seasonal viruses. The only exception is NS1 from H1N1 A/swine/Shandong/1123/2008, which is classified as A4.2, but this virus appears to have a reassortant genome, as the rest of the segments are of swine origin. This NS1 A4.2 segment has been circulating in H3N2 swine viruses for many years, as it is present in H3N2 A/swine/Ontario/00130/97 but eventually was introduced into the H1N1 A/swine/Shandong/1123/2008. Multiple other examples of segment association exist, such as 86.88% of NS1 A5.2 (217aa) being present in H9N2 Asian viruses, which is not the case in those 217aa sequences of A5.4 more associated with H6N2 viruses. In addition, 95.28% of NS1 A5.3 are associated with H3N8 segments, and 99.09% of NS1 A5.5 are associated with HPAI H5N1 segments. Other segment, host and geographical associations are likely to be found under this classification system and deserve further analyses. Of note, only three sequences showed unpaired sequence patterns to those shown in Table 3, which led to the putative identification of recent laboratory artifacts where contamination or recombination with A/Puerto Rico/8/1934 may have occurred.

Our proposed clustering method reduces the noise caused by extensive genetic drift and allowed us to identify conserved phenotypic characteristics after RNA phylogenetic tree estimations within the complex genetic background of IAV NS1. This classification of NS1 sequences clearly reveals that functional motifs previously described in strain-specific experiments are well conserved within classes, with characteristic profiles regarding functional motifs for each class.

Noronha et al. published an exhaustive analysis of Influenza Sequence Feature Variant Type (SF-VT) as a tool for genotype-phenotype association studies [42]. Based on data from the Influenza Research Database [52], we analyzed the prevalence of Influenza_A_NS1_SF33 and Influenza_A_NS1_SF30 SF-VTs corresponding to crk/crkL and PKR-binding sites, respectively, for each of the NS1 classes proposed here using the unique sequence database pdb4535 (Table 6). A characteristic SF-VT signature for each group, subgroup or lineage can be distinguished, supporting the idea that each class may also have a particular motif profile as well as phenotypes. There are some VTs shared by distant classes such as VT 9 for the PKR-binding site present in A1, A5.1, A5.6 and B (Table 6). Whether this phenomenon is a result of common ancestry or a product of convergent evolution needs to be explored.

A combination of results from phenetic and phylogenetic studies may contribute to the understanding of IAV pathogenesis and evolution, such as the identification of common phenetic traits present when sequences with different phylogenetic background infect a new host via a new route [53], [54]. We could hypothesize that the mutations required to acquire certain trait or to change host would be different depending on the segment sequence (classification) and depending on the genomic constellation of segments.

We cannot expect any particular classification to be permanent [40] while evolution continues. Our aim was to achieve the best classification with the information currently available. This updated classification was an unbiased phylogenetic classification in which the relationship between the classes is based only on the phylogenetic proximity, and we derived (phenetic) similarities for each of these classes. The number of classes (groups, subgroups, lineages) is not fixed, as it will expand or improve as new viruses are discovered and as the NS gene continues to evolve.

## Supporting Information

Figure S1 **Weblogo amino acid representation of NS1 groups, subgroups and lineages.** Protein data from unique sequences (pdb4551) was considered. Amino acids were colored as follows: acid (D, E) in red, basic (K, R, H) in blue, cysteine (C) in orange, proline (P) in purple and threonine (T) in green. The normalized height of the residue boxes indicates their relative frequency. Empty boxes indicate gaps or stop codons. Amino acid positions are marked below sequence.
(PDF)

Table S1 **Motif Profile Summary for each NS1 class.** The most frequent profile predictions for three binding motifs and the sumoylation domain are shown based on the unique sequences database (pdb4535). + and − symbols indicate inferences of predicted activity based on strain-specific studies or reverse genetics. The bar (|) notation represents the most common residues in that position in decreasing order of frequency. Each class has a characteristic motif profile, even when lineages are compared.
(DOCX)
