# Correction: Protein Clustering and RNA Phylogenetic Reconstruction of the Influenza a Virus NS1 Protein Allow an Update in Classification and Identification of Motif Conservation

**DOI:** 10.1371/annotation/e38caef3-f0a3-40d6-854a-71b545b9ddc9

**Published:** 2013-09-12

**Authors:** Edgar E. Sevilla-Reyes, David A. Chavaro-Pérez, Elvira Piten-Isidro, Luis H. Gutiérrez-González, Teresa Santos-Mendoza

The term "Influenza A" was capitalized incorrectly in the title. The correct title is: "Protein Clustering and RNA Phylogenetic Reconstruction of the Influenza A Virus NS1 Protein Allow an Update in Classification and Identification of Motif Conservation." 

The correct citation is: Sevilla-Reyes EE, Chavaro-Pérez DA, Piten-Isidro E, Gutiérrez-González LH, Santos-Mendoza T (2013) Protein Clustering and RNA Phylogenetic Reconstruction of the Influenza A Virus NS1 Protein Allow an Update in Classification and Identification of Motif Conservation. PLoS ONE 8(5): e63098. doi:10.1371/journal.pone.0063098. 

