# Supplementary material for: Protein Clustering and RNA Phylogenetic Reconstruction of the Influenza a Virus NS1 Protein Allow an Update in Classification and Identification of Motif Conservation
Source: PLoS One. 2013 May 7;8(5):e63098. doi: 10.1371/journal.pone.0063098 (PMC3646732; doi:10.1371/journal.pone.0063098)

A1

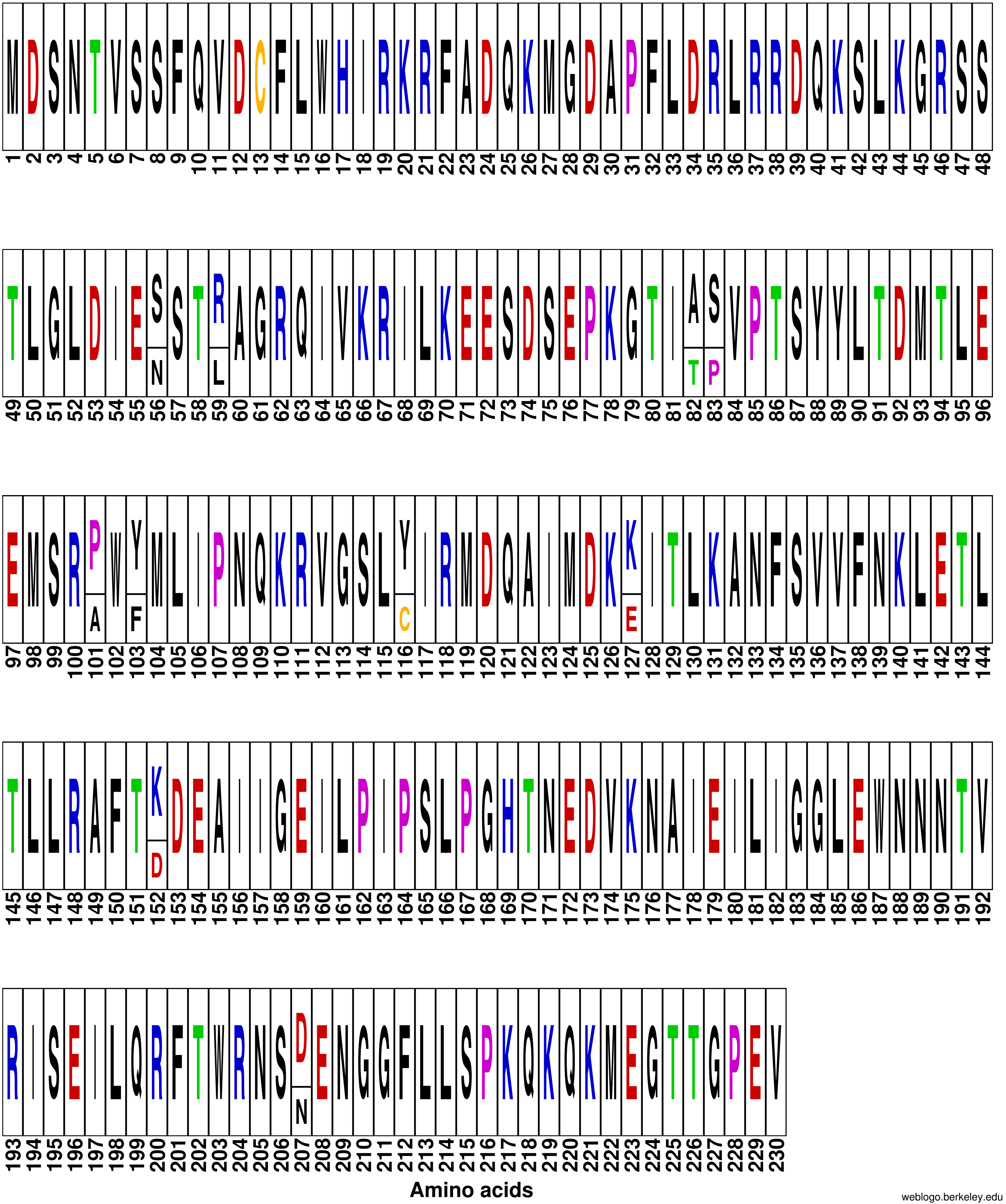

A2

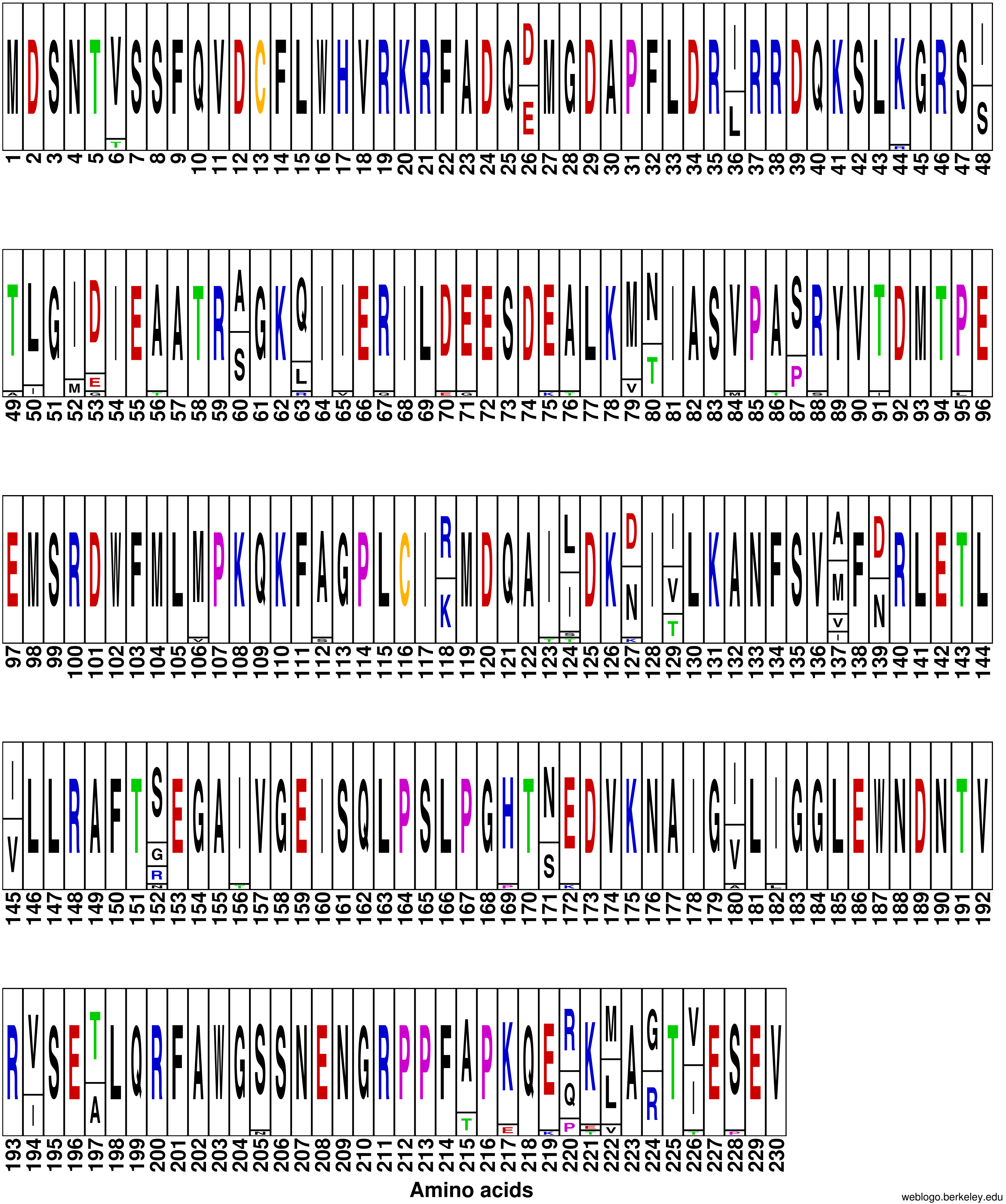

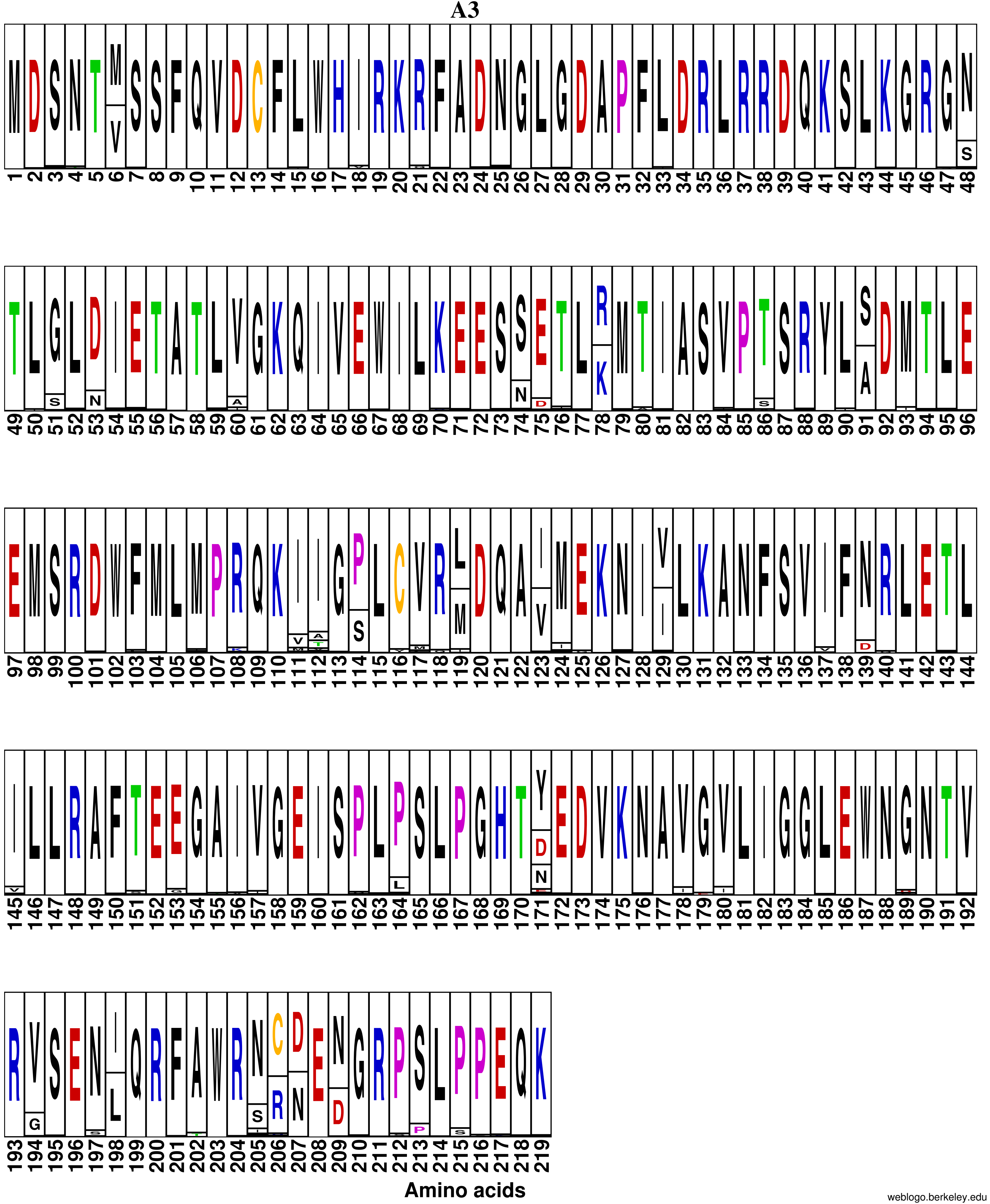
A3
Amino acids
weblogo.berkeley.edu

A4

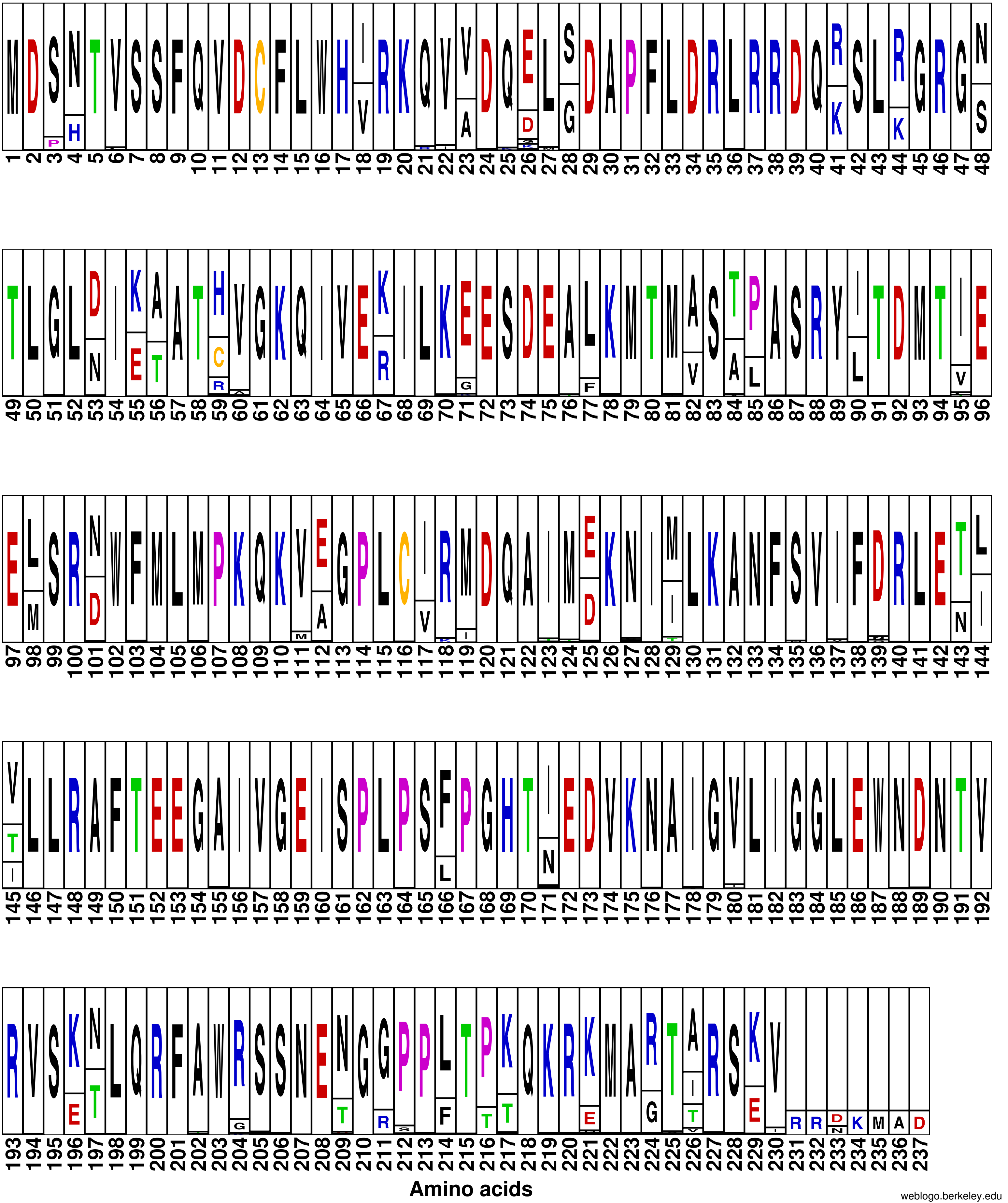

A4.1

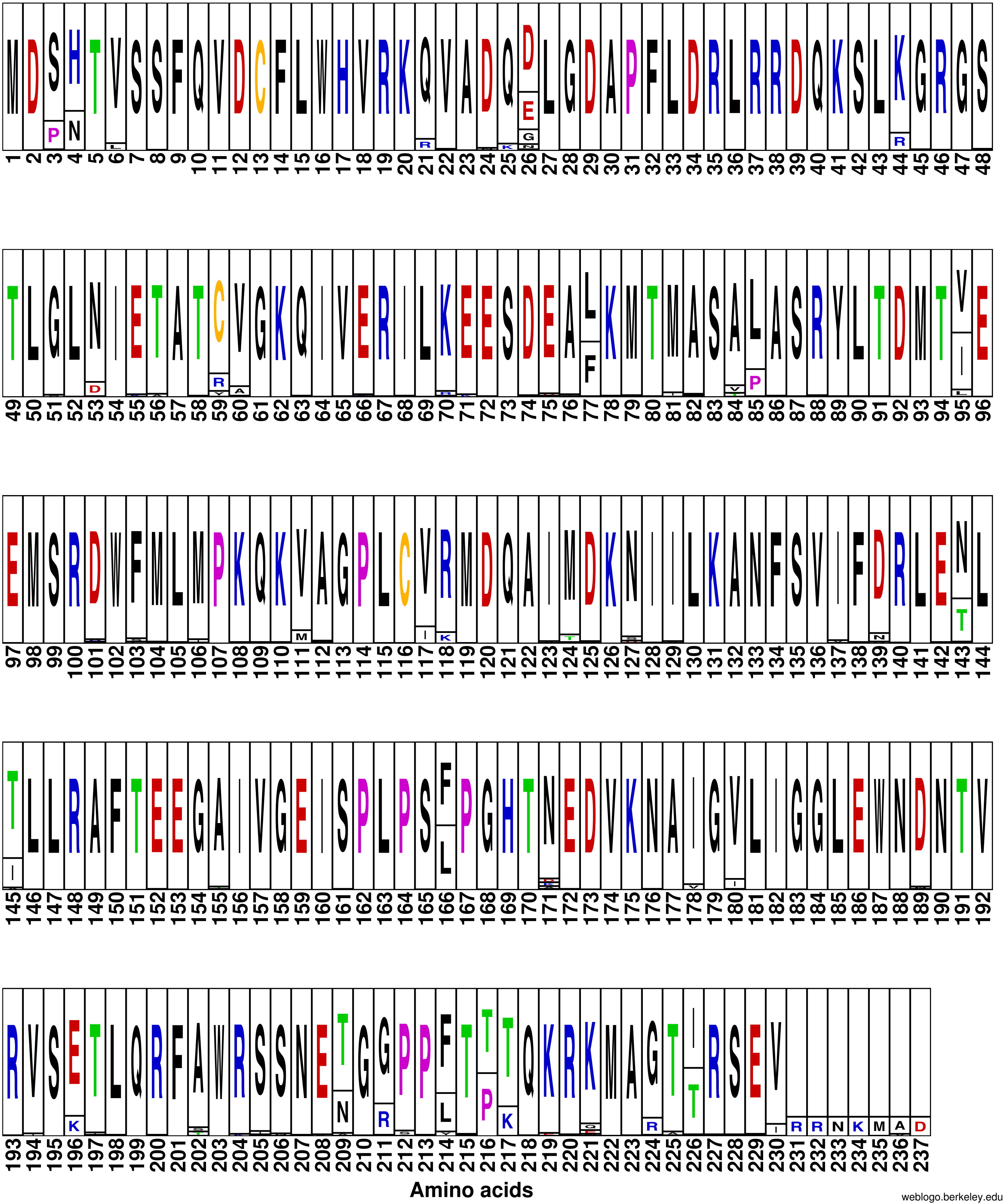

A4.2

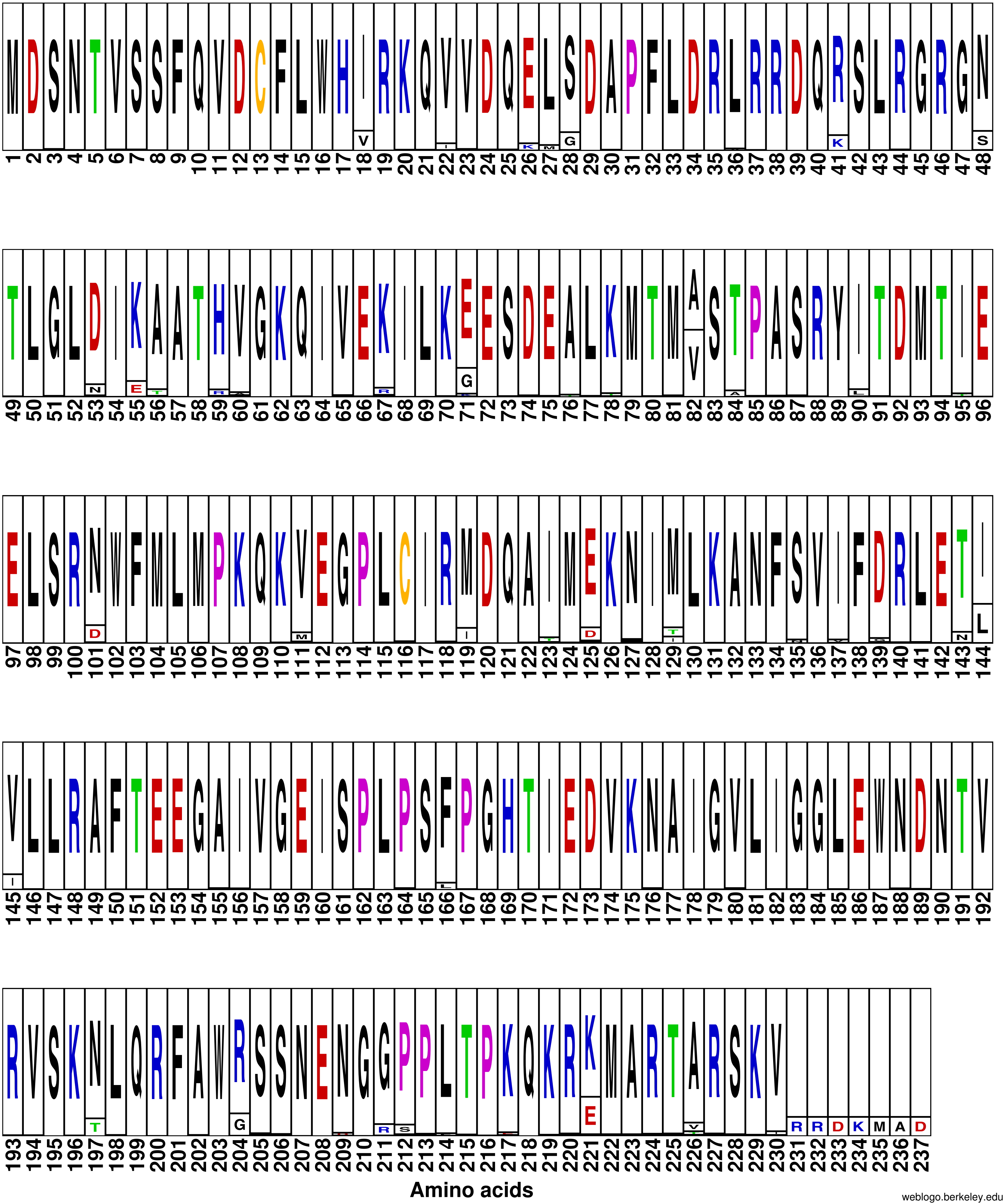

A5

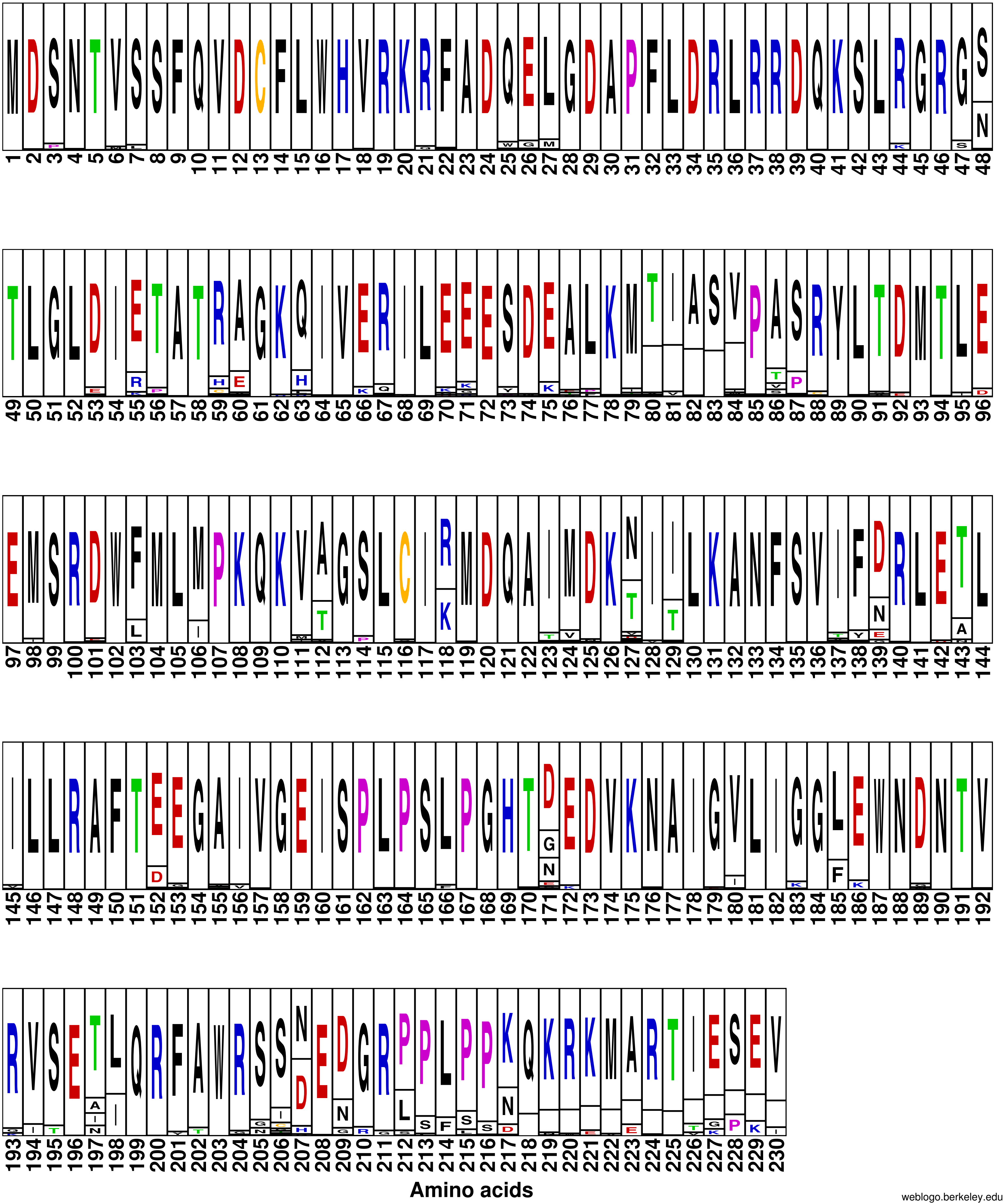

A5.1

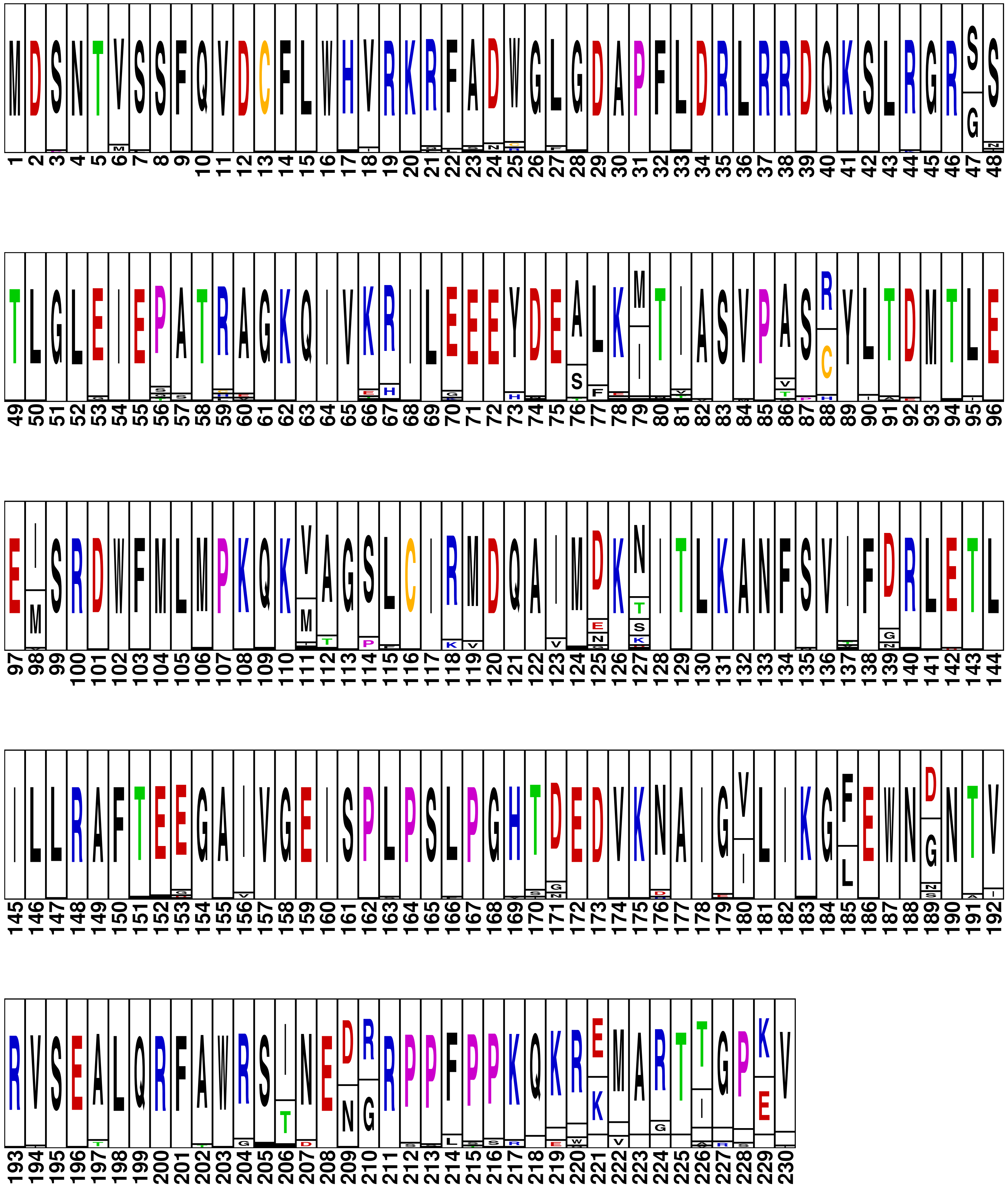

A5.2

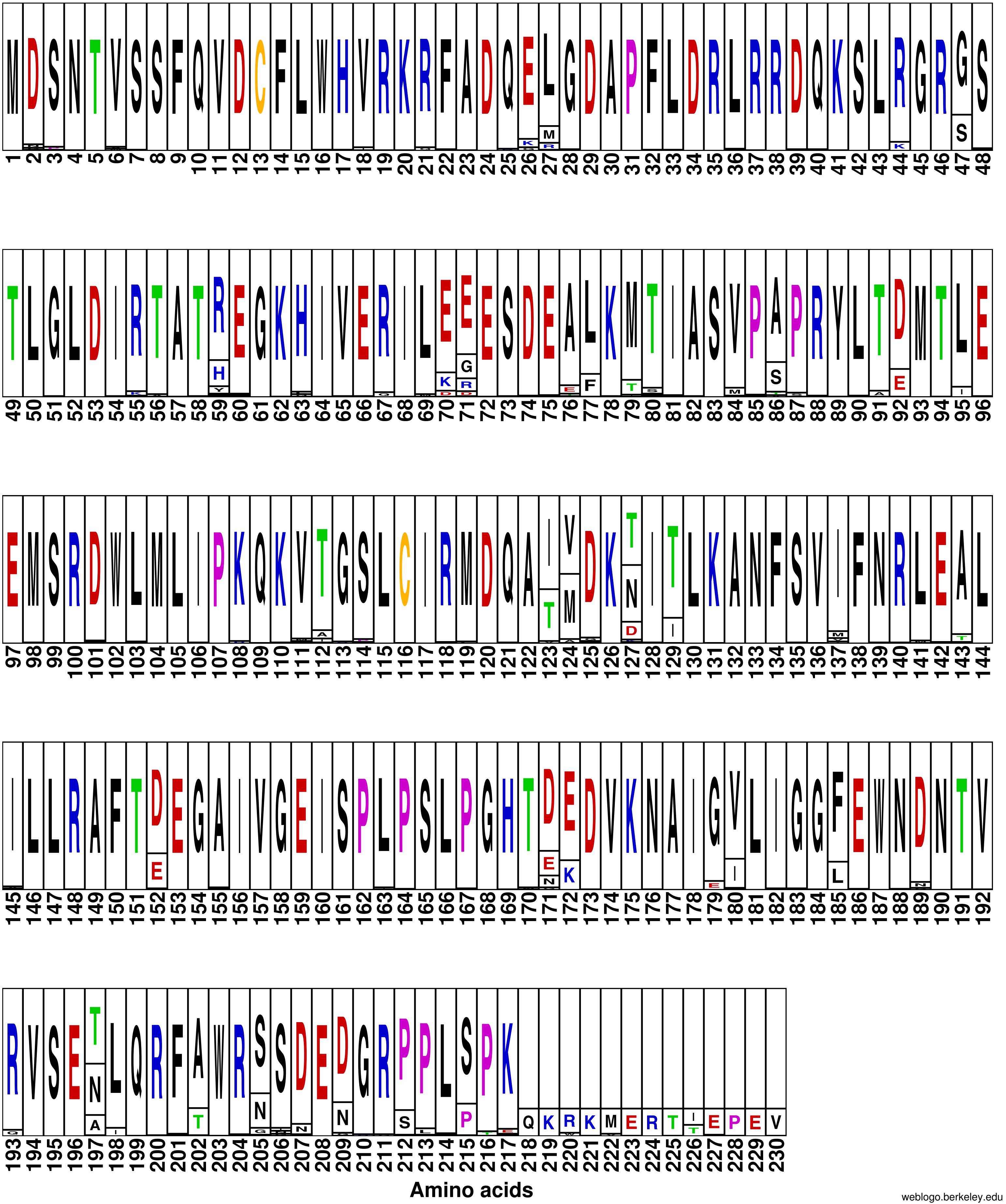

A5.3

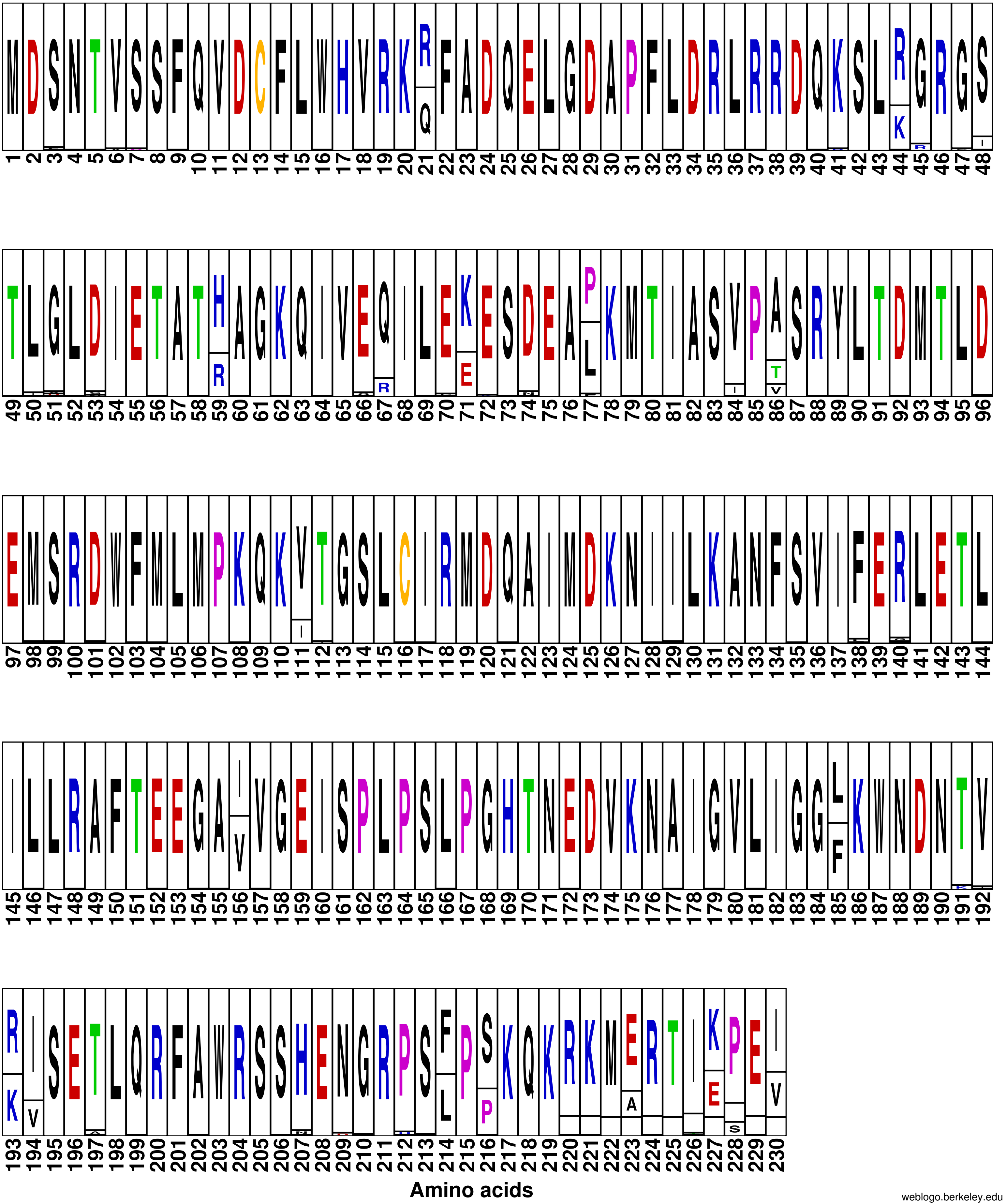

A5.4

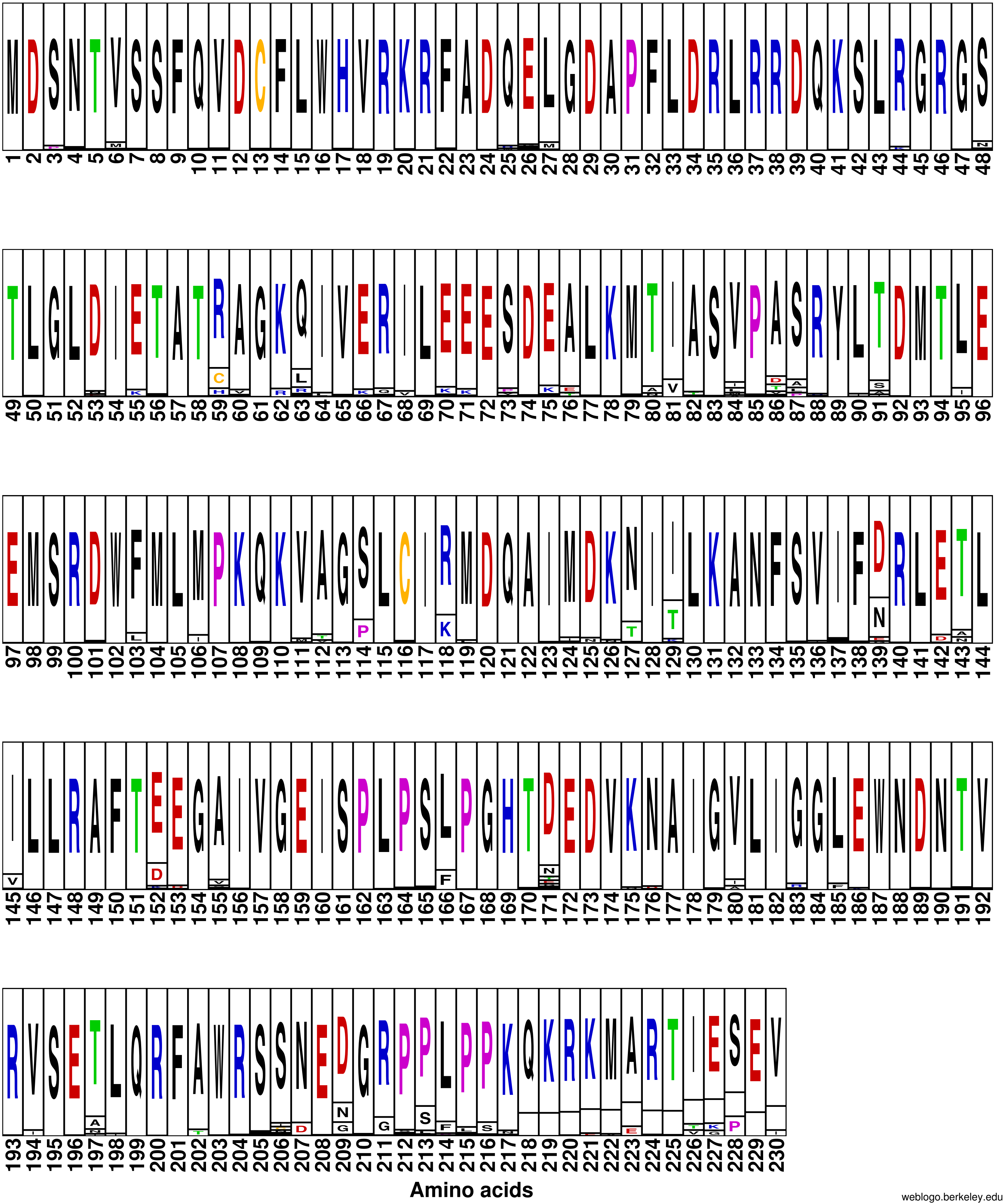

A5.5

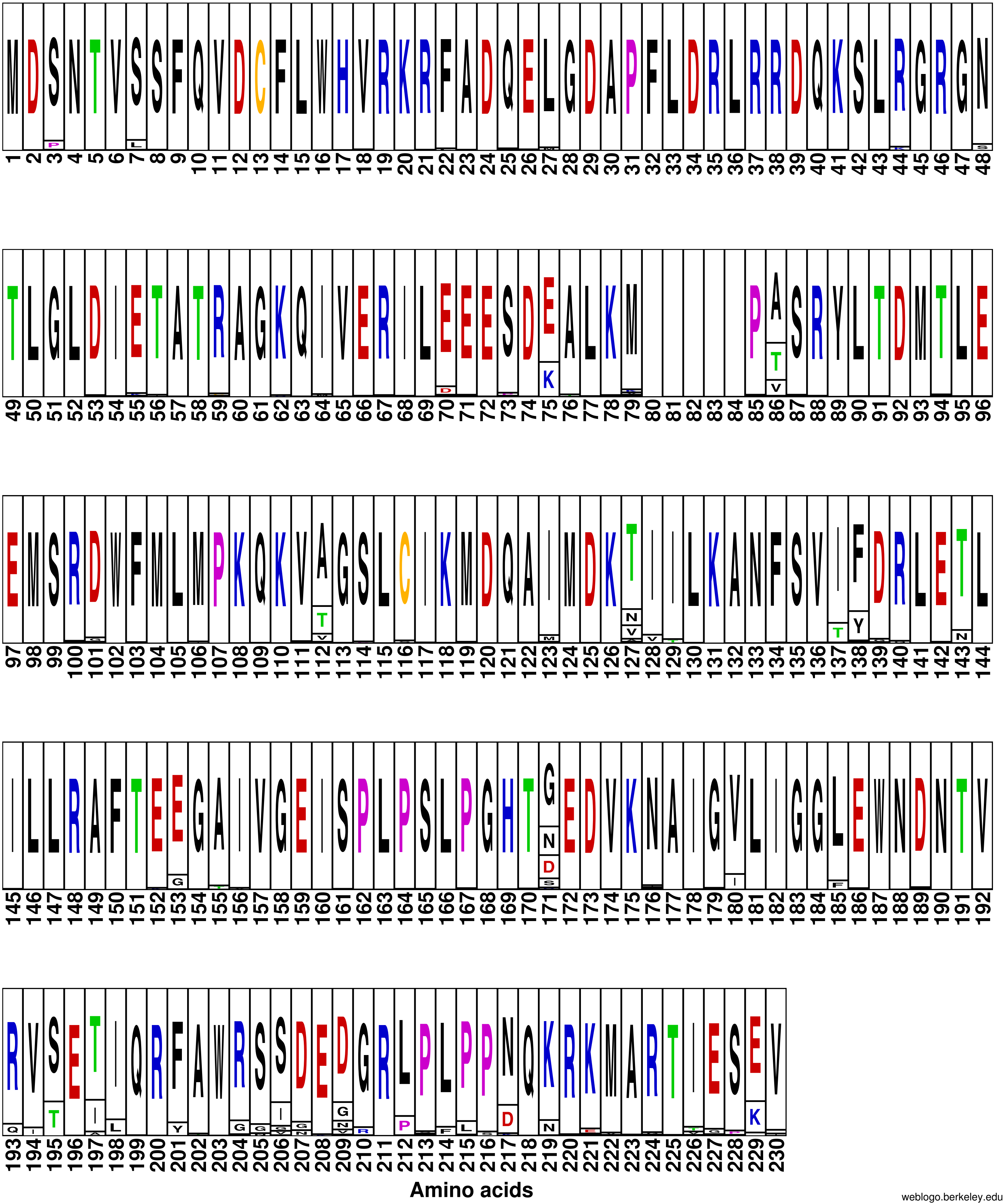

A5.6

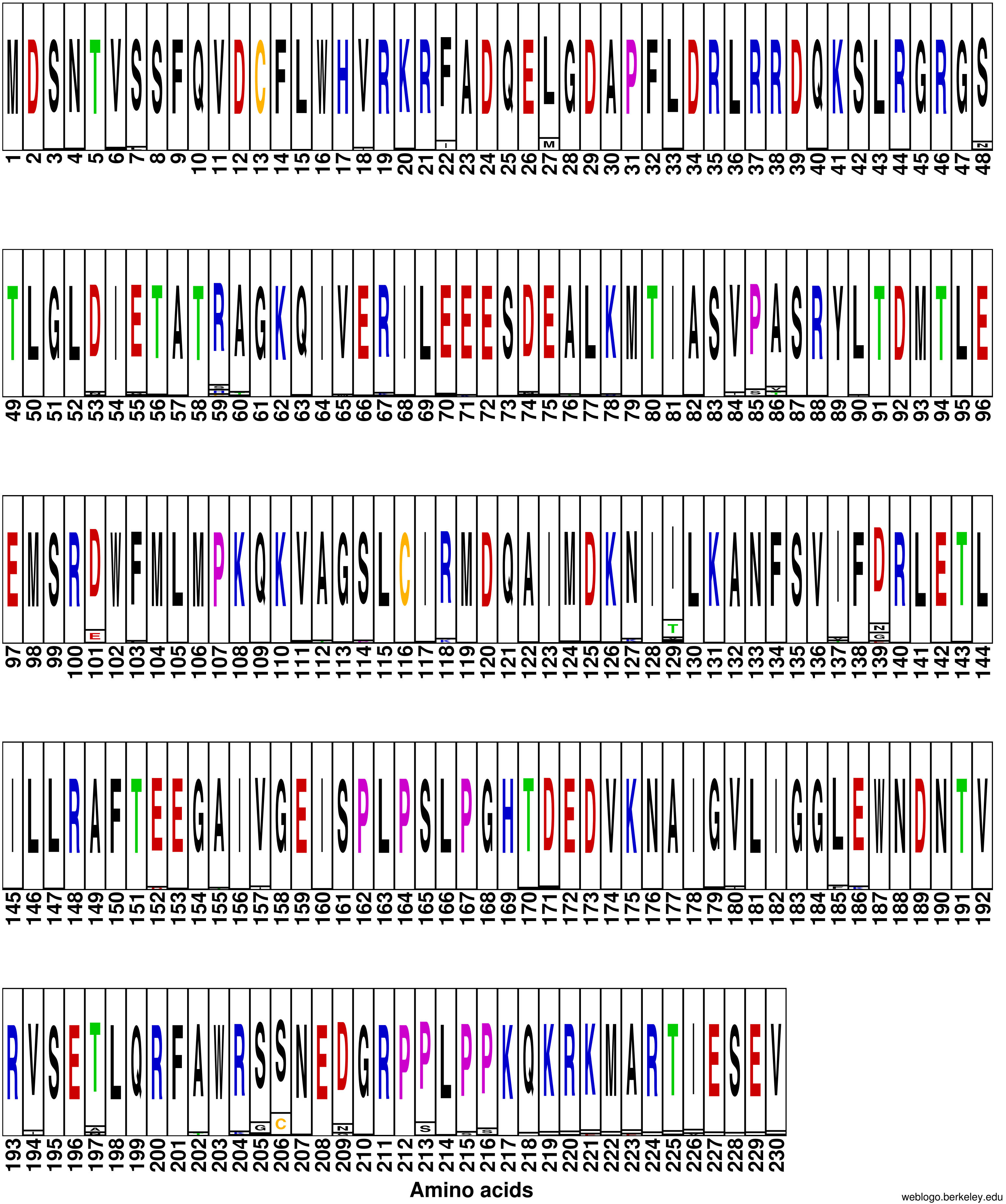

B group

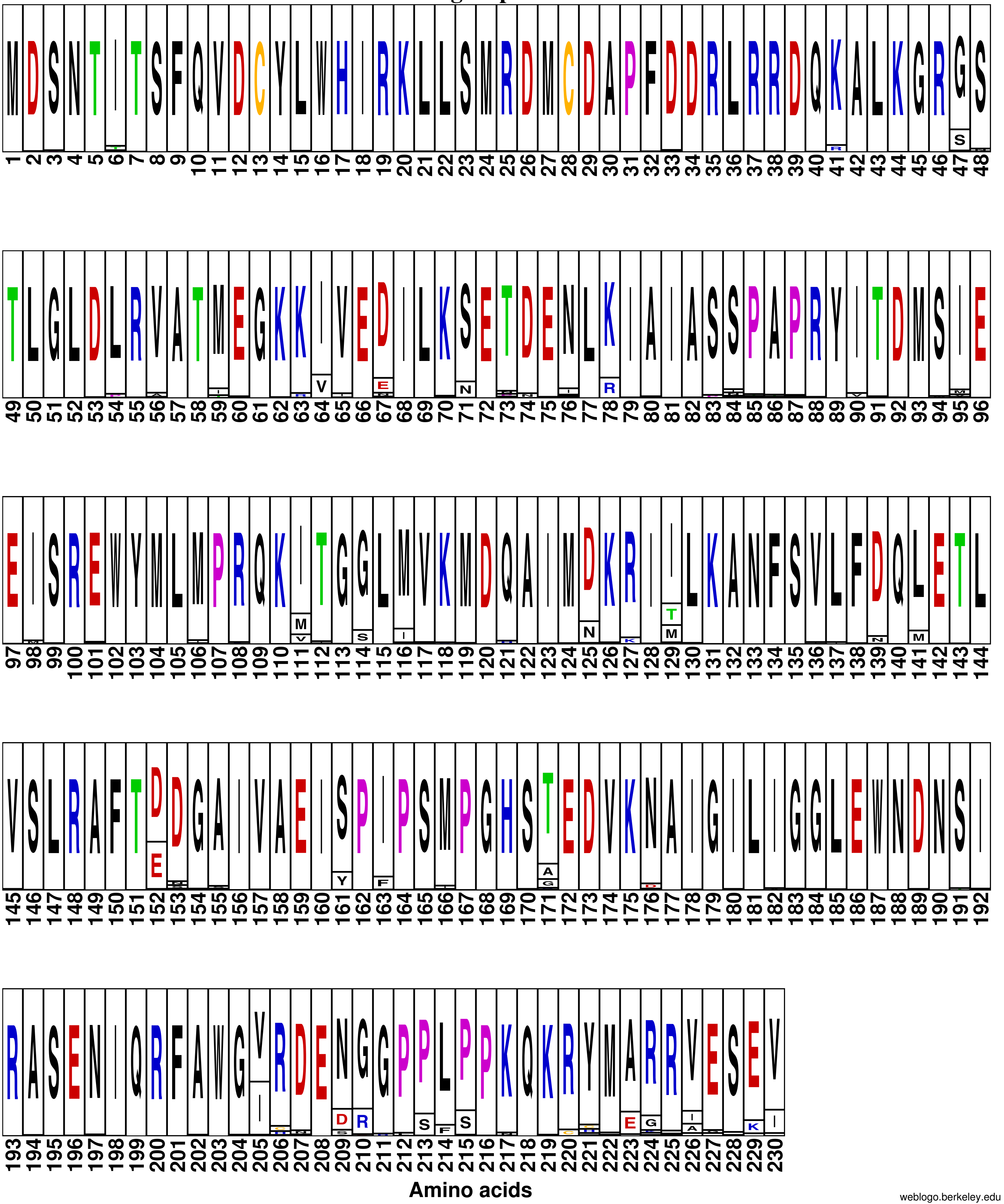

Supplement: Figure S1 — Weblogo amino acid representation of NS1 groups, subgroups and lineages. Protein data from unique sequences (pdb4551) was considered. Amino acids were colored as follows: acid (D, E) in red, basic (K, R, H) in blue, cysteine (C) in orange, proline (P) in purple and threonine (T) in green. The normalized height of the residue boxes indicates their relative frequency. Empty boxes indicate gaps or stop codons. Amino acid positions are marked below sequence. (PDF) [file pone.0063098.s001.pdf]
