# Supplementary material for: Protein Clustering and RNA Phylogenetic Reconstruction of the Influenza a Virus NS1 Protein Allow an Update in Classification and Identification of Motif Conservation
Source: PLoS One. 2013 May 7;8(5):e63098. doi: 10.1371/journal.pone.0063098 (PMC3646732; doi:10.1371/journal.pone.0063098)
Supplement: Table S1 — Motif Profile Summary for each NS1 class. The most frequent profile predictions for three binding motifs and the sumoylation domain are shown based on the unique sequences database (pdb4535). + and − symbols indicate inferences of predicted activity based on strain-specific studies or reverse genetics. The bar (|) notation represents the most common residues in that position in decreasing order of frequency. Each class has a characteristic motif profile, even when lineages are compared. (DOCX) [file pone.0063098.s002.docx]

Table S1

|  |  | **Domain** | | | | | | |
| --- | --- | --- | --- | --- | --- | --- | --- | --- |
| **Subgroup** | **Lineage** | **crk/crkLbm** | **Sumoylation** | **CPSF30bm** | **PDZbm** | | | |
|  |  |  |  |  | 227 | 228 | 229 | 230 |
| **A1** |  | - | + | - | G | P | E | V |
| **A2** |  | - | +/- | +++ | E | S | E | V |
| **A3** |  | - | - | + | - | - | - | - |
| **A4** | A4.1 | - | + | +++ | R | S | E | V |
|  | A4.2 | - | + | +++/++ | R | S | K | V |
| **A5** | A5.1 | + | + | +++/++ | G | P | K\|E | V |
|  | A5.2 (217aa) | - | - | - | - | - | - | - |
|  | A5.2 (230aa) | - | + | - | E | P | E | V |
|  | A5.3 | + | + | +++ | K\|E | P\|S | E | I\|V |
|  | A5.4 | + | + | +++ | E | S\|P | E | V |
|  | A5.5 | - | + | +++ | E | S | E\|K | V |
|  | A5.6 | + | + | +++ | E | S | E | V |
| **B** |  | + | + | - | E | S | E\|K | V\|I |
